# A Revised Framework for the Investigation of Expectation Update Versus Maintenance in the Context of Expectation Violations: The ViolEx 2.0 Model

**DOI:** 10.3389/fpsyg.2021.726432

**Published:** 2021-11-11

**Authors:** Christian Panitz, Dominik Endres, Merle Buchholz, Zahra Khosrowtaj, Matthias F. J. Sperl, Erik M. Mueller, Anna Schubö, Alexander C. Schütz, Sarah Teige-Mocigemba, Martin Pinquart

**Affiliations:** ^1^Department of Psychology, University of Marburg, Marburg, Germany; ^2^Department of Psychology, University of Leipzig, Leipzig, Germany; ^3^Center for the Study of Emotion and Attention, University of Florida, Gainesville, FL, United States; ^4^Department of Psychology, University of Giessen, Giessen, Germany

**Keywords:** ViolEx model, expectation violation, expectations, prediction error, expectation update, expectation change, expectation persistence, expectation maintenance

## Abstract

Expectations are probabilistic beliefs about the future that shape and influence our perception, affect, cognition, and behavior in many contexts. This makes expectations a highly relevant concept across basic and applied psychological disciplines. When expectations are confirmed or violated, individuals can respond by either updating or maintaining their prior expectations in light of the new evidence. Moreover, proactive and reactive behavior can change the probability with which individuals encounter expectation confirmations or violations. The investigation of predictors and mechanisms underlying expectation update and maintenance has been approached from many research perspectives. However, in many instances there has been little exchange between different research fields. To further advance research on expectations and expectation violations, collaborative efforts across different disciplines in psychology, cognitive (neuro)science, and other life sciences are warranted. For fostering and facilitating such efforts, we introduce the ViolEx 2.0 model, a revised framework for interdisciplinary research on cognitive and behavioral mechanisms of expectation update and maintenance in the context of expectation violations. To support different goals and stages in interdisciplinary exchange, the ViolEx 2.0 model features three model levels with varying degrees of specificity in order to address questions about the research synopsis, central concepts, or functional processes and relationships, respectively. The framework can be applied to different research fields and has high potential for guiding collaborative research efforts in expectation research.

## Introduction

We use expectations about us, others, and the world around us to anticipate the future and to help us navigate through our environment successfully. Expectations influence our decisions and shape our behavior in anticipation of expected experiences in many ways: Expectations help optimizing functional behaviors, from visual search and simple action execution to complex behavioral patterns when approaching rewards and avoiding threats. Stereotypical expectations about others influence how we perceive and treat people who hold different political views, belong to different ethnic or religious groups, or have physical or mental health issues. Treatment expectations influence if we adhere to psychotherapy and how much we benefit from it. Performance expectations influence how we set, adapt, and pursue our goals. In short, expectations are relevant in many areas of life and across psychological disciplines (Roese and Sherman, 2007; Hoorens, 2012; Kruglanski et al., 2020).

Theories of human perception, cognition, and action refer to making and testing predictions as the brain’s main purpose (Friston, 2009; Den Ouden et al., 2012; Clark, 2013). To improve their predictions, i.e., to minimize their prediction error^1^, individuals are able to update their expectations in response to disconfirming events, experiences, or information. Often enough, however, they do not update their expectations when violated by disconfirming evidence (Roese and Sherman, 2007; Proulx and Inzlicht, 2012; Rief et al., 2015). On the one hand, such expectation maintenance can be advantageous, for example, when disregarding probable noise (Hohwy, 2017), avoiding or attenuating negative affect after worse-than-expected experiences (Proulx et al., 2012), or protecting expectations that are relevant for values, goals, and positive beliefs that individuals hold about themselves or about the world (Greve and Wentura, 2010; Pinquart and Block, 2020). On the other hand, the maintenance or even stabilization of expectations despite disconfirming evidence – in other words, not adjusting one’s internal model of the world to reality – can have negative consequences for oneself (e.g., psychopathology; Craske et al., 2014; Kube et al., 2019, 2020) or others (e.g., stereotypes; Dort et al., 2020a,b; Kotzur and Wagner, 2021). Given the relevance of expectations and expectation violations for understanding human perception, affect, cognition, and behavior across basic and applied psychological fields, interdisciplinary accounts of predictors and mechanisms of expectation update versus maintenance are needed.

## The ViolEx Model – an Interdisciplinary Framework for Studying Expectation Update Versus Maintenance in the Context of Expectation Violations

To this point, different theoretical frameworks have aimed to model and explain the processes and outcomes associated with expectation violations, that is, how individuals respond when their expectations are disconfirmed (Pinquart et al., 2021a, for an overview). Among them, the model of Violated Expectations (ViolEx; Rief et al., 2015; Gollwitzer et al., 2018) focuses on the role of expectation violations in expectation change versus maintenance. The ViolEx model has been one of the most complete and versatile models in the sense that it refers to all classes of cognitive processes and behavioral responses to expectation violations that have been discussed across the different theories (Pinquart et al., 2021a). More precisely, it postulates cognitive mechanisms of expectation change (accommodation), different cognitive mechanisms to minimize the impact of expectation violations on expectations (data-oriented and concept-oriented immunization), as well as behaviors to actively increase the probability of future expectation confirmation and decrease the probability of expectation violation (assimilation) (Rief et al., 2015; Gollwitzer et al., 2018). The ViolEx terminology was inspired by Brandtstädter and colleagues’ model of coping with information that violates self-concepts (Brandtstädter and Greve, 1994; Brandtstädter, 2007). It is important to stress that it overlaps with those of other theories in and beyond expectation research, sometimes with similar and sometimes with different meanings (cf. Pinquart et al., 2021a). For example, the concept of accommodation by Piaget (1952) (adaptation of schemata to schema-inconsistent experiences) closely resembles accommodation in the ViolEx model. In most cases, assimilation in Piaget’s sense (fitting new experiences into schemata without changing the schemata) refers to the processing of new information that is not discrepant with a preexisting cognitive schema, although biased perceptions of discrepant information (a case of immunization according to the ViolEx model) could also lead to assimilation of new information in Piaget’s terms.

The original ViolEx model was used and evaluated in a local research training group consisting of fourteen laboratories from different psychological backgrounds, confirming the model’s potential to facilitate interdisciplinary discussion and collaboration. In this process, we also identified opportunities to further improve the model’s accessibility for researchers from different disciplines and to increase its specificity to better inform concrete research questions. We developed the ViolEx 2.0 model (ViolEx 2.0, in short) with the aims to (a) refine definitions of central constructs and their arrangement in the model where needed (also see Glossary; Table 1), (b) specify functional relationships between the different processes and moderators, and (c) elaborate on the role of behavior in expectation update versus maintenance.

**TABLE 1:** Glossary.

**Accommodation**	Mechanisms by which individuals update their *expectation* to increase consistency with the experienced *situational outcome*. Includes *expectation change* and/or *expectation (de)stabilization.*
**Approach**	Behavior influencing the situation with the goal to increase probabilities of certain *situational outcomes.*
**Assimilation**	Behavior influencing the situation with the primary goal to increase the probability of expectation-confirming *situational outcomes* and/or decrease the probability of expectation-violating *situational outcomes.*
**Avoidance**	Behavior influencing the situation with the goal to reduce probabilities of certain *situational outcomes*.
**Concept-oriented immunization**	Cognitive mechanisms by which individuals reframe the conceptual meaning of their *expectations* so that expectation-violating evidence is no longer diagnostically valid for their original *generalized expectations*.
**Data-oriented immunization**	Cognitive mechanisms by which individuals devalue expectation-violating information (e.g., ignoring or denying the information; doubting its credibility) so that it is no longer regarded as evidence against the original *expectation*.
**Expectation**	Conditional belief about the probability of future events, experiences, or information.
**Expectation change**	Change in an *expectation* with regard to what the most plausibly anticipated *situational outcomes* are.
**Expectation (de)stabilization**	Decrease or increase in confidence or certainty in an *expectation.*
**Expectation violation**	Inconsistency between situation-specific *expectation* and the internal representation of a *situational outcome*. May be specified in terms of magnitude, direction, and uncertainty.
**Experimentation**	Behavior influencing the situation with the primary goal to obtain or generate new and valid expectation-relevant information
**External anticipatory reaction**	Behavior influencing the situation to attain expectation-related goals (*assimilation* vs. *experimentation*) and/or outcome-related goals (*approach* vs. *avoidance*).
**Generalized expectation**	Abstraction of a set of more specific *expectations* with common situation and/or *situational outcome* characteristics (i.e., *expectations* about similar situations and/or similar *situational outcomes*).
**Immunization**	Cognitive mechanisms by which individuals minimize the potential impact of expectation-violating evidence on their *expectations*. It can be distinguished between *data-oriented immunization* and *concept-oriented immunization.*
**Internal anticipatory reaction**	Mostly cognitive, affective, and physiological reactions that aim at optimizing responses to expected *situational outcomes.*
**Situational outcome**	Event, experience, or information with the potential to confirm or violate an individual’s *expectations.*

**FIGURE 1:**
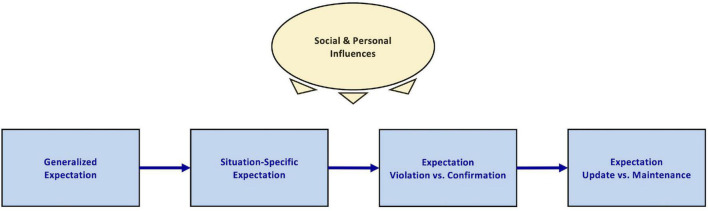
Synopsis level (Level 0) of the ViolEx 2.0 model. Overall, the model aims to inform and facilitate research on the predictors and mechanisms of expectation update and maintenance, particularly in the context of expectation violations. Generalized expectations are expectations that individuals have about groups of similar situations and their potential outcomes. Given a particular situation, generalized expectations inform more concrete situation-specific expectations. Depending on whether the outcome of a situation is consistent with an expectation or not, this expectation is confirmed or violated, respectively. Following expectation violation (or confirmation), individuals may either update their expectation to increase consistency with the situational outcome or maintain their expectation. Identical expectations, situations, and situational outcomes may still result in differences in expectation updating or maintenance across occasions and across individuals, given moderating social and personal influences.

**FIGURE 2:**
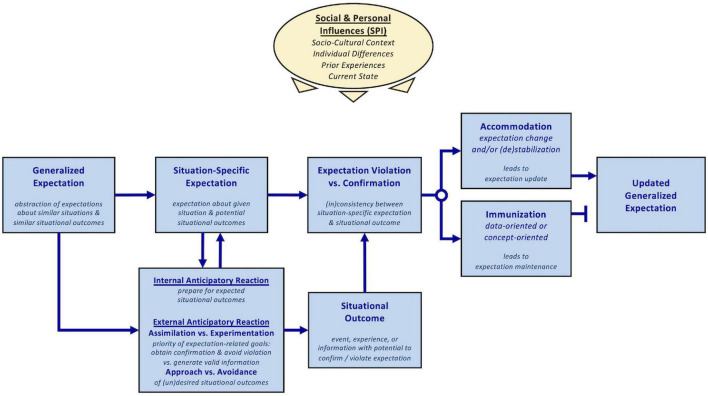
Conceptual level (Level 1) of the ViolEx 2.0 model. Generalized expectations inform situation-specific expectations. Anticipatory reactions are triggered reactively by situation-specific expectations or proactively by generalized expectations and can influence the actual or experienced situational outcome (i.e., expectation-relevant events, experiences, or information). ViolEx 2.0 distinguishes between (a) internal anticipatory reactions (typically in the attentional, cognitive-affective, or physiological domain) that aim at optimizing responses to expected situational outcomes and (b) external anticipatory reactions, that is, overt behaviors that aim at changing the probabilities of potential situational outcomes. Assimilation describes behavior to obtain or generate expectation confirmation and avoid expectation violation. Experimentation describes behavior to obtain or generate valid expectation-relevant information regardless of whether this information confirms or violates prior expectations. Approach and avoidance describe behaviors that aim to increase the probability of desirable outcomes and decrease the probability of undesirable outcomes, respectively. When external anticipatory reactions change the antecedents in the situation, the situation-specific expectation is adjusted accordingly. The comparison between expectations and situational outcomes can result in different magnitudes of expectation violation, that is, the degree of inconsistency between the situation-specific expectation and the situational outcome (no inconsistency being a case of expectation confirmation). Individuals can respond to expectation violations (or confirmations) with accommodation, that is, updating their generalized expectation by integrating the evidence, or with immunization, that is, by minimizing the situational outcome’s impact on the generalized expectation. Different social and personal influences are associated with expectation characteristics and modulate expectation-relevant processes.

**FIGURE 3:**
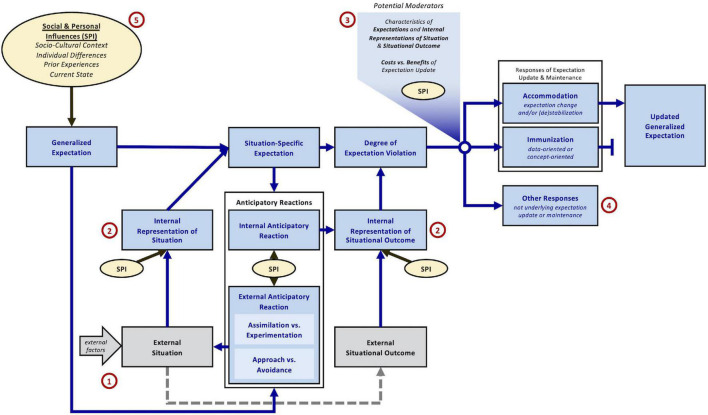
Functional level (Level 2) of the ViolEx 2.0 model. Specifications in addition to Level 1: (1) The external situation is influenced by the external anticipatory reaction and external factors outside the individual. It influences the situation-specific expectation (via the internal representation of the situation) and it ultimately leads to the external situational outcome (dashed line); (2) Distinction between the objective external situation/situational outcome (gray boxes) and their internal representations; (3) Influences on the selection of accommodation versus immunization include characteristics of the expectations, internally represented characteristics of the situation and situational outcome, potential costs versus benefits of expectation update, as well as social and personal influences [see (5)]; (4) Other responses to expectation violations that are not underlying expectation update versus maintenance; and (5) Pathways of social and personal influences (SPI) are specified: the socio-cultural context, individual differences, prior experiences, and the individual’s current state can influence generalized expectations, internal representations of situation and situational outcome, anticipatory reactions, and selection of accommodation versus immunization.

We developed ViolEx 2.0 as an open and flexible framework to guide interdisciplinary exchange and ultimately help formulating overarching research questions across fields. In order to facilitate such exchange in the first place, a framework must be accessible and provide sufficient degrees of freedom to researchers that are used to work with different field-specific models. To this end, we developed a set of general constructs and principles that experts from different fields can agree on and that allows to describe relevant mechanisms and predictors in a standardized, formalized model. The core constructs of ViolEx 2.0, which are important to explain expectation update and maintenance, have been clearly defined (Table 1) and are depicted in the diagrammatic model representation (boxes in Figures 1–3). Meanwhile, the central general relationships between these constructs are explicitly stated (arrows in Figures 1–3) providing a certain degree of formalization. To still ensure sufficient degrees of freedom for researchers from different fields, ViolEx 2.0 provides a flexible and open framework: empirical knowledge and auxiliary assumptions from other models (e.g., particular moderating variables or computational rules) can be easily located and integrated if needed. Subsequently, overarching research questions can be formulated, thus allowing (a) to probe the generalizability of field-specific principles, (b) to identify their potential boundary conditions, and (c) to eventually formulate generalized principles that can be applied across research fields.

In order to benefit from the original model’s parsimony but simultaneously achieve higher specificity in the ViolEx 2.0 model, we developed three model levels that suit different purposes and that differ in their level of detail. First, the synopsis level (Level 0) provides a short summary of the model’s scope and is well-suited for introductions and initial discussion of the topic of expectation violation (see Figure 1 for description). Second, the conceptual level (Level 1) contains all relevant psychological and behavioral constructs of ViolEx 2.0, relates them to each other, and is ideal for economically describing the entirety of processes relevant for the update and maintenance of expectations (see Figure 2). The functional level (Level 2) is a more detailed elaboration of Level 1. It specifies process dynamics and functional relationships between the ViolEx components and is suited for deducing and testing empirical hypotheses for specific parts of the model (Figure 3). The present paper is structured along the lines of the conceptual Level 1, referring to the functional Level 2 whenever appropriate. Throughout this paper, we will introduce the different model components of ViolEx 2.0 one after another. We will also show how empirical findings and more specific models from particular fields can be integrated to inform hypotheses about general mechanisms and predictors of expectation update and maintenance.

## Expectations and Expectation Violations

In ViolEx 2.0, we understand expectations as conditional beliefs about the probabilities of events, experiences, or information in the future (Roese and Sherman, 2007; Hoorens, 2012). The characteristics of a given situation (i.e., the physical and social environment relative to an individual) can be used by individuals as the antecedents of these conditional beliefs, which are used to predict situational outcomes as precisely as possible. Notably, expectations can vary in their degree of specificity for certain situations and their outcomes. *Generalized expectations* are abstractions of more specific expectations about groups of similar situations on the one hand and expected situational outcomes on the other hand (Huff and LaBar, 2010). For example, threat expectations generalize across situations when they are evoked by an entire class of cues or situations with similar attributes, not only by those that were previously followed by an aversive event (Dunsmoor and Paz, 2015; Dymond et al., 2015). As an example for generalization of outcomes, teachers who expect children with ADHD to show “typical” ADHD behavior may see their expectations confirmed when children are either distracted, impatient, or aggressive (Dort et al., 2020a). Generalized expectations are highly adaptive as they provide parsimonious heuristics about what to expect, even when situations cannot be analyzed exhaustively or contain unfamiliar elements (Gershman et al., 2015; Binz and Endres, 2019). They can be abstracted from more specific expectations (Huff and LaBar, 2010), deduced from other beliefs or premises (Johnson-Laird et al., 2015), and can be acquired via direct or indirect experiences (Olsson and Phelps, 2004). When individuals find themselves in a given situation (also see Figure 3[1]), they form *situation-specific expectations* that are derived from generalized expectations and aim at providing more precise predictions.

Both generalized and situation-specific expectations can be found in many different domains of mental processing. As a basic sensorimotor example, consider a simple reaching movement for a goal object. Human movement execution is fraught with errors, for example, endogenous motor noise. This necessitates *feedback control* (Todorov and Jordan, 2002) based on sensory signals (vision, proprioception, haptics) obtained during movement execution, if the goal is to be reached with high accuracy. However, for many swift movements, sensory processing is too slow to be useful. It has therefore been proposed that the motor system predicts the sensory consequences of its output via a *forward model* (McNamee and Wolpert, 2019). Such sensory consequences are, for example, joint configurations in proprioception, or the visually perceived hand position. Feedback corrections can then be executed based on the forward model’s predictions. This forward model is a generalized expectation: It describes the relationship between a motor command and the expected sensory consequences, for example, the final hand position after the movement. When a particular motor command is combined with information about the relative position between actor and goal object, the forward model will predict a *specific* sensory consequence, which is a situation-specific expectation, for example, the hand touching the goal object. To be useful, such predictions have to be computed quickly; thus, the forward model must have a parsimonious structure (Knopp et al., 2020).

According to ViolEx 2.0, expectations can formally be described as subjective probability distributions of potential situational outcomes. Depending on the representation of the outcomes, this distribution may be discrete (e.g., expecting that a treatment will be very effective, moderately effective, or not effective at all) or continuous (e.g., expecting to score a B on an exam with decreasing probabilities for scores that deviate more extremely from the expected value, like a D). In comparison with the original ViolEx model in which expectations were defined as “if-X-then-Y” associations between situations and situational outcomes, this conceptualization has multiple advantages: (a) it provides more degrees of freedom for researchers in operationalizing expectation-related manipulations and measures, (b) it allows to quantify uncertainty inherent in expectations, for instance, as variance or entropy of the probability distribution, and (c) it allows to characterize expectation violations more precisely (with regard to their magnitude, direction, and uncertainty; see next paragraph).

Expectations, when specific enough, can be confirmed or disconfirmed. In ViolEx 2.0, *situational outcomes* are events, experiences, or information that have the potential to confirm or violate situation-specific expectations. *Expectation violations* occur if there are inconsistencies between the perceived situational outcomes (also see *internal representation of situational outcome*, Figure 3[2]) and the original situation-specific expectations (Den Ouden et al., 2012; Clark, 2013). Depending on the representation or operationalization of expectations, expectation violations can be quantified by their magnitude (Rescorla and Wagner, 1972), direction (e.g., better-than-expected, less-than-expected, closer-than-expected; Schultz et al., 1997; Yacubian et al., 2006), and the uncertainty that is inherent in them (Feldman and Friston, 2010; Bach and Dolan, 2012; Spicer et al., 2020). As they can result from overly inaccurate expectations, expectation violations may set off an “alarm signal” when detected (Roese and Sherman, 2007) calling for attentional resources (Petersen and Posner, 2012) and corrective actions (Shadmehr et al., 2010). In the following section, we will focus on cognitive responses to expectation violations that either lead to an update of the original expectation or that prevent expectation update by minimizing the impact of the disconfirming evidence.

## Cognitive Responses to Expectation Violations: Accommodation and Immunization

In response to expectation violations, individuals can either integrate (pieces of) the disconfirming evidence into their previously held expectations and/or they can shield these expectations from such evidence. In other words, individuals can increase consistency between expectations and situational outcomes by updating their expectations and/or by re-evaluating the evidence and its implications. Individuals may show different degrees of immunization after expectation-violating outcomes (e.g., ignoring some vs. all discrepant information) associated with different degrees of accommodation (e.g., stronger vs. weaker expectation change).

### Accommodation

*Accommodation* includes mechanisms by which individuals update their expectations following expectation violation (or confirmation) to increase consistency with the experienced outcome. Expectation updates can include expectation changes in case of expectation violations (i.e., the outcomes that are expected as most plausible change in the direction of the disconfirming outcome) and expectation (de)stabilization (i.e., the confidence or certainty in an expectation decreases or increases). For example, in motor control, accommodation can occur when a motor output’s actual sensory consequences do not match the expected sensory consequences and when this mismatch is attributed to an error in the expectation. Consider the reaching example described earlier: if the hand misses the goal object, then the actor might accommodate their expectations about the sensory consequences of the motor output, that is, adapt the forward model. This accommodation does not require conscious choice: it has been demonstrated that there are implicit (automatic) and explicit (consciously accessible) components to movement adaptation (Schween and Hegele, 2017). The implicit component likely counteracts the effects of continuous changes of the human body’s biomechanical properties throughout the day, for example, muscle fatigue or limb length changes. These changes would lead to expectation violations in the forward model.

Functional and computational accounts of accommodation mechanisms can be found in classical (Rescorla and Wagner, 1972; Pearce and Hall, 1980) or Bayesian accounts of associative learning (Kruschke, 2008; Gershman, 2015), in which prediction errors (i.e., expectation violations) are a necessary condition for expectation change. In ViolEx 2.0, the accommodation concept has been broadened compared to the original ViolEx model. In the original model, accommodation was equated with “learning.” It occurred exclusively in response to expectation violation (but not confirmation) and incorporated only expectation change but not expectation (de)stabilization. Accommodation now refers to any case in which experienced situational outcomes are integrated into expectations. Thereby, the revised concept of accommodation is compatible with learning accounts that model degrees of expectation violation continuously without a (potentially arbitrary) categorical distinction between expectation violation and confirmation. It is also compatible with the notion that changes in confidence or uncertainty (i.e., destabilization and stabilization) can be understood as instances of learning as well (e.g., Bayesian belief updating; Chater et al., 2006).

### Immunization

*Immunization* refers to a class of mechanisms that aim at minimizing the impact of evidence disconfirming the original expectation and thereby prevent expectation update. *Data-oriented immunization* influences the evaluation of the experienced situational outcome. This includes ignoring or denying information (Nickerson, 1998), assigning low reliability or credibility to the information source (Dunn and Schweitzer, 2005; Sarathchandra and Haltinner, 2020), and subtyping (categorizing the outcome as exception to the rule; Carnaghi and Yzerbyt, 2007; Riek et al., 2013). Examples for data-oriented immunization after violated motor control expectations include unsuccessful reaching for an object in a nearly dark room (failure might be attributed to low reliability of the visual input) or trying to hit a fly (action would have been successful if the fly had not moved as quickly). Hence, a forward model update can be omitted. Meanwhile, in *concept-oriented immunization*, the conceptual meaning of a situation-specific expectation is reframed so that it becomes irrelevant for the protected generalized expectation (Greve and Wentura, 2010). For example, a person may have their expectations about the hostility of an outgroup violated when encountering an outgroup member who smiles at them. Instead of updating their expectation or denying the smile, however, the first person might reason *post hoc* that the smile is just a superficial response intended to hide hostile feelings inside or that it represents a mean smile (cf. Paulus et al., 2016). In other words: the violated expectation about the outgroup member’s overt behavior has become diagnostically irrelevant for probing the generalized expectation that members of said outgroup (secretly) hold hostile feelings.

### Influences on Responding With Accommodation or Immunization

Whether individuals respond to expectation violations with accommodation and/or immunization depends, among other factors, on the magnitude of the expectation violation. Small violations often are neglected (immunization) because readily adjusting expectations to noise comes with more cognitive costs than benefits (Gershman et al., 2015; Hohwy, 2017; Kruglanski et al., 2020). Beyond such negligible expectation violations, the “delta rule” predicts that higher magnitudes of expectation violations will result in higher magnitudes of expectation change, a facet of accommodation (Rescorla and Wagner, 1972). However, when expectation violations become extremely large, beyond some threshold, individuals may engage in subtyping (i.e., immunization) and categorize their experiences as exceptions to the rule (Summerfield and Tsetsos, 2015; Filipowicz et al., 2018) or doubt the validity of their priors (Niv, 2019). This suggests that the relationship between expectation violation magnitude and probability of accommodation may follow an inverted U-shape (Filipowicz et al., 2018) rather than a monotonously increasing function. However, at this point, more systematic research is needed to identify boundary conditions (i.e., why has the delta rule been appropriate for associative learning but not for violations of stereotypes) and to probe the plausibility of any generalized formal rule (e.g., the U-shaped relationship). Here, ViolEx 2.0 has been developed to address these very questions. It guides the identification of relevant field-specific principles and provides the frame in which these principles can be communicated to other researchers so that new studies can be conceptualized. In addition to the magnitude, the direction of expectation violation may influence cognitive responses; for instance, in optimism biases, with stronger immunization tendencies for unpleasant compared to pleasant expectation violations (Sharot et al., 2011; Garrett and Sharot, 2017). Again, ViolEx 2.0 can be used to investigate whether stronger immunization tendencies to unpleasant expectation violations generalize *per se* or under which conditions they do.

In terms of utility maximization, the probability of accommodation or immunization also depends on the costs and benefits of expectation updates (Figure 3[3]). As previously pointed out, the cognitive costs of a more complex generalized expectation may outweigh the benefit of slightly more accurate predictions that result from it (Kruglanski et al., 2020). Alternatively, integrating expectation violation experiences without increasing expectation complexity (i.e., more complicated premises) can only be achieved by increasing uncertainty levels in expectations (i.e., less precise outcome predictions; Kwisthout et al., 2017) – which can be experienced as aversive in itself (Grupe and Nitschke, 2013; Pepperdine et al., 2018). Psychological costs of expectation update may also come from having to accept inconvenient truths about oneself (Wilson and Dunn, 2004; Greve and Wentura, 2010; Korn et al., 2012) or the world (Sharot et al., 2011). Moreover, expectation updates may be associated with social benefits or costs when they are (not) aligned with those of relevant peers (Esser, 1998; Hogg and Hains, 1998; Kuntsche et al., 2014; Robertson and Tustin, 2018).

In addition to accommodation and immunization, ViolEx 2.0 now explicitly includes other responses to expectation violations that arise from processes that are not the basis of expectation update or maintenance (Figure 3[4]). Among them are, for example, immediate attentional (e.g., increase in alertness, orienting; Petersen and Posner, 2012), corrective (e.g., adaptations in motor control; Shadmehr et al., 2010), or palliative responses (e.g., committing to other, non-violated expectations; Proulx et al., 2012), that aim at avoiding or buffering unwanted consequences of expectation violations. Although these other responses are not mechanistically involved in expectation update or maintenance, they can be of interest for researchers, for example, as indicators of expectation, outcome, or expectation violation characteristics (e.g., explicit appraisals of expectation strength, surprise, or disappointment; Sweeny and Dillard, 2014; Körfer et al., 2020).

## Influencing Actual and Experienced Situational Outcomes: Anticipatory Reactions

Individuals do not passively await situations and their outcomes but instead actively select and modify the situation. ViolEx 2.0 describes *anticipatory reactions* that depend on situational outcomes expected by individuals and that aim either at preparing for these outcomes or at actively changing outcome probabilities. Anticipatory reactions may be triggered by situation-specific expectations (i.e., reaction to situation characteristics) or be shown proactively motivated by generalized expectations (i.e., proactive selection of situation characteristics).

For ViolEx 2.0, we significantly expanded the concept of anticipatory reactions relative to the original model, in order to achieve our goal of elaborating on the role of behavior in expectation updating versus maintenance. As we will describe in more detail in the following paragraphs, ViolEx 2.0 distinguishes between internal anticipatory reactions to *prepare* for anticipated situational outcomes and external anticipatory reactions to *influence* situational outcome probabilities. Moreover, external anticipatory reactions can be characterized by expectation-related goals (assimilation vs. experimentation behavior) and by outcome-related goals (approach vs. avoidance behavior; also see Figure 2).

### Internal Anticipatory Reactions

*Internal anticipatory reactions* aim at optimizing responses to expected outcomes. Other than external anticipatory reactions (see below), they do not aim at changing the situation and associated situational outcome probabilities, but rather influence how the situational outcome will be experienced (internal representation of the situational outcome, Figure 3). Internal anticipatory reactions include, for example, anticipatory affective states (Davis et al., 2009) and physiological changes of behavioral activation or inhibition (Krause et al., 2018; Hashemi et al., 2019). Of particular importance, they also include attention modulations that may result in heightened selective attention to predictive cues (Miskovic and Keil, 2012; Panitz et al., 2019) and optimized attention guidance toward expected outcome locations (Bergmann et al., 2019, 2020). Anticipatory attention modulation supports the detection and integration of expectation-relevant information and enhances processing of prediction error signals (Kok et al., 2012; Jiang et al., 2013; Smout et al., 2019) thereby facilitating adaptive accommodation responses to expectation violations. In turn, however, effective attention guidance itself depends on reliable and precise expectations about relevant information (Torrents-Rodas et al., 2021) and uncertain expectations may result in hypervigilance (Wieser et al., 2016; Kastner-Dorn et al., 2018). Importantly, attention processes can also be biased, as seen, for instance, in threat biases (Aue and Okon-Singer, 2015; Koenig et al., 2017), confirmation biases (Nickerson, 1998; Kappes et al., 2020), or optimism biases (Kress and Aue, 2017), which skew the probability of accommodation versus immunization responses. While additional assumptions from field-specific models are needed to predict which type of bias will be shown in which situations, ViolEx 2.0 allows to integrate these assumptions and level the existing knowledge about biases to investigate their role in mechanisms of expectation update and maintenance.

### External Anticipatory Reactions

*External anticipatory reactions* refer to overt behaviors that aim at actively influencing the situational circumstances and thereby ultimately changing situational outcome probabilities (Figure 3). These behaviors can be further characterized based on their expectation- and outcome-related goals.

Assimilation and experimentation describe behaviors that are motivated by expectation-related goals. More specifically, *assimilation* behavior aims at generating or obtaining situational outcomes that confirm one’s expectations (which may be realistic or not; Miller and Turnbull, 1986; Bessi et al., 2015; Del Vicario et al., 2016; Hechler et al., 2016) and avoid situational outcomes that violate them (Klayman and Ha, 1987; Charpentier et al., 2018; Sharot and Sunstein, 2020). In contrast, *experimentation* refers to behavior aimed at generating or obtaining valid expectation-relevant situational outcomes, not biased toward confirming prior expectations. In general, motivation to assimilate or experiment depends on the potentially obtainable information, which can have both inherent (cognitive or hedonic) and instrumental value (Gottlieb et al., 2013; Sharot and Sunstein, 2020). Feedback-regulated motor control (Todorov and Jordan, 2002) is a prime example of *assimilation*: in the above-mentioned reaching example, an actor regulates their motor output toward the goal, with the objective of avoiding an expectation violation. However, before one can assimilate successfully, one must first have valid generalized expectations (forward models). “Body babbling” observed in human infants might be considered as *experimentation* with the goal of developing such valid expectations (Meltzoff and Moore, 1997).

Drawing on previous findings on information seeking and processing, we formulated hypotheses about general predictors of assimilation and experimentation behavior. For example, ViolEx 2.0 assumes that both assimilation and experimentation can be used to reduce uncertainty in expectations or at least avoid increases in uncertainty (cognitive value). Here, previous research suggests that high degrees of uncertainty inherent in an expectation primarily favor experimentation (Lee et al., 2011; Mehlhorn et al., 2015). First, the benefits of increasing the precision of simple and uncertain expectations typically outweigh the costs of increased complexity (Gershman et al., 2015; Kruglanski et al., 2020). Second, unelaborated expectations simply do not provide specific outcome expectations that individuals are biased to confirm. In addition, higher instrumental value of information (i.e., the information’s utility for optimizing goal-directed behaviors; Gottlieb et al., 2013) increases motivation for experimentation because adaptive instrumental behavior requires expectations that closely reflect real-world contingencies. Meanwhile, assimilative strategies should become more likely when uncertainty is lower and it becomes more effective to reduce or limit uncertainty by selectively generating expectation-confirming or avoiding expectation-disconfirming information (Klayman and Ha, 1987; Nickerson, 1998). In addition, assimilation for positively valued expectations should become more likely when potential information has a high (positive or negative) hedonic value. Under this assumption, pleasant anticipated information will increase the motivation to obtain confirming evidence while unpleasant anticipated information will increase the motivation to avoid disconfirming evidence (Scherer et al., 2012). Notably, the overall information value – and therefore the motivation to engage in assimilation or experimentation – would result from the total of information value facets (Sharot and Sunstein, 2020). Future systematic research is needed to test whether these assumptions can be formalized (cf. Sharot and Sunstein, 2020) in a way that they are universally valid across research contexts and/or what additional boundary conditions need to be specified.

The concepts of assimilation and experimentation share some overlap with the concepts of information seeking (in the case of assimilation and experimentation) as well as exploration (in the case of experimentation). Building upon the original ViolEx model, we chose to keep the label *assimilation* and add *experimentation* for the following reasons. First, information seeking often is associated with conscious and deliberate behavior to obtain new explicit knowledge, for example, when patients search the internet for information about symptoms or treatments (Zhao and Zhang, 2017). While assimilation and experimentation include such information seeking strategies, they also include instances in which individuals act upon their environment to create expectation confirmation without deliberately seeking for new knowledge (e.g., behavior in the context of self-fulfilling prophecies). Second, experimentation in ViolEx 2.0 is closely related to the general concept of exploration. Nonetheless, we chose the label *experimentation* in order to avoid confusion with some exploration conceptualizations that are incompatible with our experimentation concept, for example, because they do not refer to open behavior (e.g., visual exploration; Mirza et al., 2018; Rubo et al., 2020) or assume random selection of behavioral alternatives (Sutton and Barto, 1998).

The second dimension described under external anticipatory reactions, *approach* versus *avoidance*, describes the degree to which behavior is motivated by outcome-related goals. Motivation for approach or avoidance depends on the subjective value of a desired or undesired situational outcome itself (Corr, 2013) rather than the outcome’s implications for an expectation. Approach behaviors aim to influence the situation in order to increase the probability of expected outcomes (typically associated with positive subjective values) while avoidance behaviors aim to decrease the probability of expected outcomes (typically with negative subjective value). Although approach and avoidance behaviors are not defined by expectation-related goals in ViolEx 2.0, they can play a central role in expectation update and maintenance. A prominent example is fearful avoidance (Mowrer, 1956; Bouton et al., 2001; Vlaeyen and Linton, 2012; Benke et al., 2019). Here, individuals will avoid situations because of an anticipated situational outcome’s inherent aversiveness (e.g., pain) which prevents exposure to potential correcting expectation violations (i.e., no pain) and, as a consequence, leads to stable and invalid generalized expectations about threat. These dynamics are reflected on the functional Level 2 of ViolEx 2.0 (Figure 3), where successful avoidance behavior terminates the threat situation before it plays out and transforms it into a safe situation. This modification of the situation (a) prevents violation of threat expectations and (b) evokes situation-specific safety expectations that are subsequently confirmed (Lovibond et al., 2009; LeDoux et al., 2017).

A necessary condition for the execution of external anticipatory responses is a minimum sense of control about the situation (sometimes also referred to as sense of agency) of the acting individual (e.g., Karsh and Eitam, 2015). Here, it is less important whether the situation is *objectively* controllable but rather whether the agent feels or judges that an outcome can be influenced by them. On the one hand, prolonged experiences of low control may result in experiences of “helplessness” where goal-directed behavior is suppressed (Seligman, 1972) and individuals may become less sensitive to changes in action-outcome contingencies (Soral et al., 2021). Deviations of experienced control from reality imply that there is no valid internal representation of the objective situation (cf. Level 2 of ViolEx 2.0) and anticipatory reactions can become dysfunctional. For example, aversive outcomes may not be avoided even though it is possible. On the other hand, individuals may have illusions of control and overestimate the influence that they have on a given situation (Moore, 2016). Illusions of control have been associated with superstitious beliefs (Griffiths et al., 2019), for example, in ineffective gambling strategies (Henslin, 1967; Griffiths, 1994). Whether sense of control is perceived – and perceived accurately – depends both on dispositional (Rotter, 1966; Bryant, 1989) and situational factors (see Thompson et al., 1998; Farrer et al., 2013; Yarritu et al., 2014, for a review). While an in-depth discussion of these factors goes beyond the scope of the present paper, they may be incorporated in research questions addressed within the ViolEx framework. Finally, sense of control can be conceptualized as expectations about action-outcome contingencies (Friston et al., 2013). This means that ViolEx 2.0 cannot only incorporate high versus low sense of control to predict the likelihood of anticipatory responses; it can also be used to investigate mechanisms of updating or maintaining said control expectations in the same way it can be used for any other type of expectation.

Compared to the original ViolEx model, ViolEx 2.0 significantly expands the concept of anticipatory reactions, specifically external anticipatory reactions. First, assimilation was relocated within the model. The original ViolEx model listed assimilation behavior as an immediate response to expectation violation, together with the cognitive mechanisms of accommodation and immunization. In ViolEx 2.0, assimilation is described as behavior that is mostly proactive or motivated by *impending* expectation violations rather than directly triggered by preceding expectation violations. Nonetheless, experiencing expectation violations may lead to accommodation which, in turn, can motivate assimilation. For example, when expectation violations lead to destabilization (accommodation), assimilation is a potential strategy to restore confidence. Second, in contrast to the original ViolEx model, ViolEx 2.0 allows to describe behavior independently on dimensions of expectation-related goals (i.e., that directly aim at updating or maintaining expectations in a particular way) and outcome-related goals (that, nonetheless, can lead to expectation update or maintenance indirectly as a byproduct). That does not mean, however, that the dimensions of assimilation versus experimentation and approach versus avoidance are necessarily exclusive. For example, a student may study hard to get a good grade, both motivated because the good grade is rewarding in itself (approach) and because it confirms the student’s expectation of doing smart things and writing good grades in general (assimilation).

## Social and Personal Influences

ViolEx 2.0 postulates that different *social and personal influences* (socio-cultural context, individual differences, prior experiences, current state) are associated with characteristics of generalized expectations. Moreover, they influence and moderate processes and behaviors related to expectation update or maintenance so that objectively comparable situations lead to different behavior and responses to expectation violations across occasions and individuals (Figure 3[5]). First, social and personal influences affect internal representations of the situation and the situational outcome. On the one hand, they modulate what characteristics of the real-world situation and its outcome we perceive and how we experience them. On the other hand, current internal states may be used as expectation premises as well (e.g., having a feeling about something to happen). Second, social and personal influences moderate anticipatory reactions by influencing the ability and motivation to show different behaviors. Third, they influence the probability (and magnitude) of expectation update or maintenance in response to expectation violations. In the following paragraphs, we will elaborate on these mechanisms with some examples. For a more comprehensive review, see Pinquart et al. (2021b).

### Socio-Cultural Context

The *socio-cultural context* includes relatively stable social circumstances like norms and other influences from society, media, and peers. Social norms provide information about the utility of individual expectations and behaviors (i.e., external anticipatory reactions; Hawkins et al., 2019). They can serve as simple and efficient heuristics (Legros and Cislaghi, 2020), especially when expectations are uncertain (Higgs, 2015). Public opinion and social climate can have a significant impact on individuals’ expectations. For example, negative expectations about immigrants and diversity among right-wing authoritarians are stronger in countries with more prominent multicultural ideologies (Kauff et al., 2013) and in countries with a negative collective view on immigrants (Cohrs and Stelzl, 2010). Generalized expectations that have been acquired via socio-cultural norms will, in turn, influence the experience of individuals’ environment (i.e., internal representations of situations and situational outcomes), for example, when stereotypical expectations about outgroups inform situation-specific expectations about particular outgroup members (Unkelbach et al., 2008).

Media coverage or statements from public persons can also influence individual expectations, for example, expectations about symptoms and abilities of individuals with mental or somatic health issues (Benbow, 2007; Miller, 2007; Pearl et al., 2012), expectations about gender differences in professional skills or sexuality (ter Bogt et al., 2010; Steinke, 2017), or expectations about the threat from ethnic or religious outgroups (e.g., Muslims in Western countries; Ogan et al., 2014). Previous studies suggested that media coverage primarily impacts individuals with congruent prior expectations (Crandall et al., 2018; Stürmer et al., 2019), providing most evidence for expectation confirmation and stabilization as central mediators. Notably, effects of media coverage or public opinion on expectations are more effective in individuals who have no or limited direct experiences with the expectation topic (or group) to confirm or disconfirm their expectations (Liebe et al., 2018).

In a narrower social context, research has highlighted the significant influences of peers on expectations. For example, higher exposure to peers with more positive attitudes toward alcohol (Cumsille et al., 2000; Kuntsche et al., 2014) or smoking (Romer and Hennessy, 2007; Urbán, 2009) predicts more positive expectations in adolescents about the experience and consequences of alcohol and tobacco consumption, respectively. First, peers’ expectations may have direct influences on generalized expectations, that is, individuals adopt their peers’ standards for orientation. Second, peers can provide opportunities to make experiences to confirm peer expectations. For example, making pleasant experiences with alcohol will lead to according expectation accommodation, potentially leading to repeated deliberate consumption (i.e., approach) and stabilization of these appetitive expectations (further accommodation). Third, accommodating expectations to match peer expectations may come with social benefits (e.g., social inclusion; Kuntsche et al., 2014).

### Individual Differences

The model’s social and personal influences further include *individual differences* like personality traits and other stable personal dispositions (e.g., genes). At this point, there is primarily evidence for personality traits that are relevant for specific expectation contents. For example, negative affective traits (e.g., neuroticism, trait anxiety), are associated with stable, negative expectations about the probability and consequences of threats as well as low self-efficacy expectations to cope with these threats (Barlow et al., 2014). Several processes that can be incorporated into ViolEx 2.0 contribute to this stability such as attentional threat biases (internal anticipatory reaction; Aue and Okon-Singer, 2015), repeated and more generalized avoidance behavior (external anticipatory reaction; Lommen et al., 2010), and interpretation biases (immunization; Hirsch et al., 2016) that facilitate maintenance of expectations even after better-than-expected outcomes.

Another prominent trait is optimism, which is defined by stable positive expectations that generalize to many different situations and situational outcomes (Scheier and Carver, 1985). Increased stability of positive expectations likely stems from stronger immunization tendencies, although it is not clear yet whether enhanced immunization in optimistic individuals occurs selectively to worse-than-expected outcomes (Geers et al., 2010; Sharot et al., 2011) or to expectation violations in general, regardless of valence (Geers and Lassiter, 2002; Morton et al., 2011). Other dispositions in the positive affectivity spectrum, like agentic extraversion or trait (an)hedonia, may influence accommodation and/or immunization tendencies as well, as they have been shown to modulate processing of situational outcomes (here, feedback processing; Mueller et al., 2014, 2015). In line with this assumption, it has been repeatedly shown that individuals suffering from major depressive disorder, who have low levels of agentic extraversion and trait hedonia (Mueller et al., 2015), show stronger immunization responses after better-than-expected situational outcomes (Kube et al., 2017, 2019, 2020).

Another group of relevant traits are cognitive styles and preferences: Originally, higher levels of need for cognitive closure (i.e., the desire for clear and non-ambiguous answers) (Kruglanski and Webster, 1996) and need for structure (i.e., the preference for simple mental models; Neuberg and Newsom, 1993; Neuberg et al., 1997) have been conceptualized to indicate preferences for simple and categorial mental models. In line with this concept, higher trait levels were shown to facilitate immunization after violations of stereotypical expectations (Dijksterhuis et al., 1996) and assimilation strategies before decision making (Hart et al., 2012). Recent findings, however, suggest that these effects are context-dependent and actually may reverse (i.e., individuals show lower tendencies for immunization and higher tendencies for experimentation) when (a) categorial expectations have not yet been established and (b) there is more disconfirming than confirming information available (Kemmelmeier, 2015).

To this point, the roles of personality traits in expectation-relevant cognitions and behaviors have been investigated in separate lines of research without exchange between the fields. Although it is theoretically plausible that broad meta-traits exist (DeYoung, 2015) that predict accommodation or immunization tendencies across expectation types as well as external anticipatory reactions (Hirsh et al., 2009), this has not been tested systematically yet (Pinquart et al., 2021b).

### Prior Experiences

*Prior experiences* refer to an individual’s learning history about situation-outcome associations and experiences with previously shown expectation-relevant responses or behaviors. First, higher numbers of prior experiences with (similar) situations typically lead to more confidence about the resulting generalized expectations which increases their stability (Roese and Sherman, 2007). However, the effect of prior experiences may not only depend on their sheer number but also on their heterogeneity: In simple classical conditioning studies, expectations that have been acquired with intermittent pairing (i.e., the cue is sometimes, but not always, followed by the outcome) are more resistant to extinction (i.e., accommodation after expectation violation) than expectations acquired with continuous pairing (i.e., the cue is always followed by the outcome; “partial reinforcement extinction effect”; Haselgrove et al., 2004; Chan and Harris, 2017). From an associative learning perspective, the continuous pairing leads to higher associative strength between cue and outcome which ultimately results in a larger prediction error and stronger reduction in associative strength when the outcome is unexpectedly omitted. Therefore, at least for very simple cue-outcome associations, expectations that are based on a homogeneous set of prior experiences could be assumed to be particular vulnerable to single expectation violations. As pointed out earlier, however, expectations may be maintained even in response to strong expectation violations (immunization). First, undesirable expectation changes may be avoided by subtyping the experience. Second, unexpected situational outcomes may be associated with other cues present in the situation (Rescorla, 1969; Kutlu and Schmajuk, 2012; Sosa and Ramírez, 2019) or with contextual information (Lucke et al., 2014; Uengoer et al., 2020b). This, in turn, allows individuals to infer that the unexpected outcome resulted from other latent causes (Niv, 2019) than previously assumed (i.e., the premises in the situation were incorrect), such that the original expectation remains unchanged.

Previous effective or ineffective attempts of accommodation, immunization, assimilation, experimentation, avoidance, or approach can influence self-efficacy and expectations about the responses’ effectiveness. This then influences the probability to show similar responses in the future (Bandura, 1977, 1982). Furthermore, at least for repeated worse-than-expected violations of highly self-relevant expectations, there might be a prototypical order of coping attempts: at first, individuals will tend to immunize. If immunization is not successful, assimilation attempts will be undertaken given that self-efficacy expectations are sufficiently strong. If assimilation strategies fail in avoiding further expectation violations – or are deemed non-effective in the first place – individuals will resort to accommodation (Brandtstädter and Greve, 1994; Pinquart et al., 2021b).

### Current State

Lastly, we have added *current state* as an influence to ViolEx 2.0, referring, for example, to transient mental, emotional, and physiological states of an individual. For example, higher cognitive load leads to shifts from proactive to reactive anticipatory reactions (Mäki-Marttunen et al., 2019), interferes with detection of outcome information (Savage et al., 2019) and thorough outcome evaluation (Hamamouche et al., 2018), and increases sensitivity for expectation-violating information (Sherman et al., 1998). Alertness and arousal can influence internal anticipatory reactions via attentional modulation (Oken and Salinsky, 1992; Vogt et al., 2008); and arousal can facilitate more sustained accommodation (Sharot and Phelps, 2004; Mickley Steinmetz et al., 2012). Pharmacological manipulations may alter physiological and mental states and may thereby, for example, influence anticipatory reactions (Willadsen et al., 2018; Berz et al., 2021) or evaluation of situational outcomes (Mueller et al., 2011, 2014).

Emotional states may influence and moderate different expectation-related processes as well. For example, it has been repeatedly shown that negative affective states (e.g., sadness) lead to higher expectations about future negative mood states (“affective forecasting”; Hoerger et al., 2012; Marroquín et al., 2016), a potential mechanism in upholding pessimistic expectations in depressive disorder (Marroquín and Nolen-Hoeksema, 2015). Here, the current emotional state may constitute a situational cue itself (influencing the internal representation of the situation; Schwarz and Clore, 2003) that is predictive of a situational outcome (i.e., feeling sad now is a predictor of feeling sad later). Notably, affective states also influence likelihood estimates of events that are not logically or causally related to one’s emotional state, for example, the likelihood of being killed by tornadoes (Johnson and Tversky, 1983), the likelihood that oneself will be honored for a major achievement (Hoerger et al., 2012), or the likelihood that someone else will get rejected by a person they love (DeSteno et al., 2000). In other words, emotional states may directly shift generalized expectations in an emotion-congruent direction to be more optimistic or pessimistic. Moreover, emotional states can influence attentional processes (i.e., internal anticipatory reaction) like the processing of global versus local attributes in positive versus negative mood states (Gasper and Clore, 2002).

## The ViolEx Model and Other Frameworks on Expectations and Expectation Violations

The ViolEx model is not the only framework that addresses questions of expectation violation, update, and maintenance. We recently compared the ViolEx model and six other frameworks (Pinquart et al., 2021a), which can be grouped with regard to their scope: First, there are broad, universal frameworks like the Meaning Maintenance Model (Proulx et al., 2012) and a social psychological model of expectancy and expectancy disconfirmation (Roese and Sherman, 2007). Second, some models are of particular relevance to specific components of ViolEx 2.0, like associative learning theories (accommodation; e.g., Rescorla and Wagner, 1972; Pearce and Hall, 1980) or the predictive coding framework (accommodation and assimilation; Schultz et al., 1997; Friston, 2009). Lastly, some frameworks have been developed for specific research content like the Expectancy Violation Theory of social interactions (Burgoon, 1983) or the Expectation-Disconfirmation Theory of consumer satisfaction (Oliver, 1980, 2014). For a detailed description and comparison of these frameworks, we refer readers to Pinquart et al. (2021a).

In general, ViolEx 2.0 is among the models with the broadest scope of applications. It addresses both short-term and long-term dynamics of expectation updates or maintenance rather than just immediate responses to expectation violations. Moreover, ViolEx 2.0 addresses the role of behavior more thoroughly than any other framework, particularly by defining the different classes of external anticipatory reactions. Finally, ViolEx 2.0 provides a more detailed description and formalized assumptions about (a) which classes of moderators (i.e., social and personal influences) affect expectation update or maintenance and (b) via which mechanisms they exert their effects. Besides these general advantages of ViolEx 2.0, there are several key differences between our model and different other models that may inform a researcher’s choice of framework. On the one hand, the diagrammatic representation of ViolEx 2.0 is more formalized than other broad, verbal frameworks (i.e., the Meaning Maintenance Model or the social psychological model by Roese and Sherman). On the other hand, models from associative learning and predictive coding theories can provide even higher degrees of formalization, including computational rules of expectation update. Moreover, if research questions are narrower or address consequences of expectation violations other than expectation update versus maintenance, content-specific models (i.e., Expectancy Violation Theory of social interactions, Expectation-Disconfirmation Theory of consumer satisfaction) might be more tailored to researchers’ needs. Nonetheless, all of the mentioned frameworks can easily be integrated within ViolEx 2.0 to combine their content specificity with the ViolEx model’s broad and interdisciplinary scope.

The main focus of ViolEx 2.0 is the description and explanation of expectation maintenance and update. While Level 2 of the ViolEx 2.0 acknowledges responses to expectation violations that are not underlying expectation update or change, other models address this class of responses in more detail: The Meaning Maintenance Model, for example, emphasizes the goal of reducing aversive arousal created by violated expectations. This includes mechanisms that are not underlying the update or maintenance of violated expectations. Moreover, in their social psychological model, Roese and Sherman suggested “tagging” as a relevant mechanism in delayed expectation update. Here, expectation violations may be acknowledged but they do not lead to accommodation right away. Instead, memories of expectation violation episodes are “tagged onto” the relevant expectations and retrieved in future occurrences of comparable expectation violations. However, the literature on the tagging concept is scarce and is primarily focused on memory encoding and retrieving rather than on mechanisms of expectation update (McClelland et al., 1995; Klein et al., 2002). As of now, delayed effects of expectation violations on expectation update can be modeled in ViolEx 2.0 as an immediate destabilization that makes expectations more prone to change after recurring expectation violation. Nonetheless, future research may address the plausibility of tagging, ideally in an interdisciplinary setting.

## Open Questions

The ViolEx 2.0 model in its current form provides a comprehensive interdisciplinary framework for studying expectation maintenance and change in the context of expectation violations, yet several aspects are left open. First, the current classification of social and personal influences is rather descriptive than functional. While there is an enormous number of empirical findings that can be readily integrated into ViolEx 2.0, the long-term goal will be to develop more generalized principles about which social and personal influences facilitate which behaviors and cognitive responses. This, in turn, will allow the ViolEx model itself to make more precise predictions and to become more testable. As an example, the following assumption could be formulated and tested: social or personal factors (e.g., values, goals, peer opinions, etc.) that are relevant for expectations related to one’s self-concept may predict the motivation to protect these expectations and thereby a higher likelihood of assimilation and immunization (cf. Greve and Wentura, 2010). However, more systematic, interdisciplinary research is needed to generate such generalized principles and probe their validity. Here lies the great potential of ViolEx 2.0, which provides the framework to guide these very research efforts.

Second, ViolEx 2.0 implies that all changes in anticipatory reactions following expectation violations are mediated via accommodation: an expectation is changed and/or (de)stabilized, which causes changes in behavior. It needs to be debated and empirically tested whether modeling a direct influence from expectation violations on future anticipatory reactions is needed to describe and explain psychological and behavioral phenomena not captured by the current model.

Third, the model does not address explicitly the processes involved in the generalization from situation-specific expectation violations to generalized expectations (Huff and LaBar, 2010; Niv, 2019; Uengoer et al., 2020a). It is plausible to assume that relationships between generalized and specific expectations are more complex than currently modeled. For example, a certain situation-specific expectation may be informed by more than one generalized expectation or individuals may be able to access a hierarchy of superordinate and subordinate expectations with different degrees of generalization.

## Conclusion

In the present paper, we introduced the ViolEx 2.0 model, an interdisciplinary framework for studying predictors and mechanisms of expectation update and maintenance, particularly in the context of expectation violations. Building upon a previous version (Rief et al., 2015; Gollwitzer et al., 2018), our goal was to design a model that can be used flexibly in collaborative research across research fields. To this end, the developed model contains three levels with different purposes and different degrees of specificity. The three levels combine parsimonious descriptions of expectation-relevant ideas and concepts with concrete descriptions of functional relationships that are necessary to derive testable hypotheses. Moreover, ViolEx 2.0 elaborates on the role of behavior in the updating and maintenance of expectations and explicitly distinguishes between objective situations and situational outcomes on the one side and their internal representations on the other side. It can easily be applied to research fields across psychology, cognitive (neuro)science, and other life sciences and will allow researchers from different disciplines to collaborate effectively. ViolEx 2.0 thereby can be an important tool for a comprehensive advancement of our knowledge on the acquisition, change, and persistence of expectations as well as the role of expectation violations therein.

## Data Availability Statement

The original contributions presented in the study are included in the article/supplementary material, further inquiries can be directed to the corresponding author.

## Author Contributions

All authors developed the model together under CP lead. CP created the figures and wrote the first manuscript draft. All authors provided critical revisions of the manuscript.

## Conflict of Interest

The authors declare that the research was conducted in the absence of any commercial or financial relationships that could be construed as a potential conflict of interest.

## Publisher’s Note

All claims expressed in this article are solely those of the authors and do not necessarily represent those of their affiliated organizations, or those of the publisher, the editors and the reviewers. Any product that may be evaluated in this article, or claim that may be made by its manufacturer, is not guaranteed or endorsed by the publisher.

## Footnotes

1 We will treat *expectation* and *prediction* as well as *expectation violation* and *prediction error* as synonyms, respectively.

## References

[B1] Aue T. Okon-Singer H. (2015). Expectancy biases in fear and anxiety and their link to biases in attention. *Clin. Psychol. Rev.* 42 83–95. 10.1016/j.cpr.2015.08.005 26379081

[B2] Bach D. R. Dolan R. J. (2012). Knowing how much you don’t know: A neural organization of uncertainty estimates. *Nat. Rev. Neurosci.* 13 572–586. 10.1038/nrn3289 22781958

[B3] Bandura A. (1977). Self-efficacy: Toward a unifying theory of behavioral change. *Psycholog. Rev.* 84 191–215. 10.1037/0033-295X.84.2.191 847061

[B4] Bandura A. (1982). Self-efficacy mechanism in human agency. *Am. Psychol.* 37 122–147. 10.1037/0003-066X.37.2.122

[B5] Barlow D. H. Ellard K. K. Sauer-Zavala S. Bullis J. R. Carl J. R. (2014). The origins of neuroticism. *Perspect. Psycholog. Sci.* 9 481–496. 10.1177/1745691614544528 26186755

[B6] Benbow A. (2007). Mental illness, stigma, and the media. *J. Clin. Psychiatry* 68 31–35. 17288505

[B7] Benke C. Krause E. Hamm A. O. Pané-Farré C. A. (2019). Predictors of behavioral avoidance during respiratory symptom provocation. *Behav. Res. Therapy* 112 63–67. 10.1016/j.brat.2018.11.012 30502722

[B8] Bergmann N. Koch D. Schubö A. (2019). Reward expectation facilitates context learning and attentional guidance in visual search. *J. Vision* 19 1–18. 10.1167/19.3.10 30916725

[B9] Bergmann N. Tünnermann J. Schubö A. (2020). Reward-predicting distractor orientations support contextual cueing: Persistent effects in homogeneous distractor contexts. *Vision Res.* 171 53–63. 10.1016/j.visres.2020.03.010 32408054

[B10] Berz A. Pasquini de Souza C. Wöhr M. Schwarting R. K. W. (2021). Limited generalizability, pharmacological modulation, and state-dependency of the habituation towards pro-social 50-kHz calls in rats. *iScience* 2021:102426. 10.1016/j.isci.2021.102426 33997703 PMC8102916

[B11] Bessi A. Coletto M. Davidescu G. A. Scala A. Caldarelli G. Quattrociocchi W. (2015). Science vs conspiracy: collective narratives in the age of misinformation. *PLoS One* 10 1–17. 10.1371/journal.pone.0118093 25706981 PMC4338055

[B12] Binz M. Endres D. (2019). Where do heuristics come from? *CogSci* 2019 1402–1408.

[B13] Bouton M. E. Mineka S. Barlow D. H. (2001). A modern learning theory perspective on the etiology of panic disorder. *Psycholog. Rev.* 108 4–32. 10.1037/0033-295x.108.1.4 11212632

[B14] Brandtstädter J. (2007). *Das flexible Selbst: Selbstentwicklung zwischen Zielbindung und Ablösung [The flexible self: Self-development between target commitment and detachment].* Amsterdam: Elsevier.

[B15] Brandtstädter J. Greve W. (1994). The aging self: Stabilizing and protective processes. *Dev. Rev.* 14 52–80. 10.1006/drev.1994.1003

[B16] Bryant F. B. (1989). A four−factor model of perceived control: avoiding, coping, obtaining, and savoring. *J. Person.* 57 773–797. 10.1111/j.1467-6494.1989.tb00494.x

[B17] Burgoon J. K. (1983). Interpersonal expectations, expectancy violations, and emotional communication. *J. Lang. Soc. Psychol.* 12 30–48. 10.1177/0261927X93121003

[B18] Carnaghi A. Yzerbyt V. Y. (2007). Subtyping and social consensus: The role of the audience in the maintenance of stereotypic beliefs. *Eur. J. Soc. Psychol.* 37 902–922. 10.1002/ejsp.402

[B19] Chan C. K. J. Harris J. A. (2017). Extinction of Pavlovian conditioning: The influence of trial number and reinforcement history. *Behav. Proc.* 141 19–25. 10.1016/j.beproc.2017.04.017 28473250

[B20] Charpentier C. J. Bromberg-Martin E. S. Sharot T. (2018). Valuation of knowledge and ignorance in mesolimbic reward circuitry. *Proc. Natl. Acad. Sci. U. S. A.* 115 E7255–E7264. 10.1073/pnas.1800547115 29954865 PMC6077743

[B21] Chater N. Tenenbaum J. B. Yuille A. (2006). Probabilistic models of cognition: Conceptual foundations. *Trends Cogn. Sci.* 10 287–291. 10.1016/j.tics.2006.05.007 16807064

[B22] Clark A. (2013). Whatever next? Predictive brains, situated agents, and the future of cognitive science. *Behav. Brain Sci.* 36 181–204. 10.1017/S0140525X12000477 23663408

[B23] Cohrs J. C. Stelzl M. (2010). How ideological attitudes predict host society members’ attitudes toward immigrants: exploring cross-national differences. *J. Soc. Issues* 66 673–694. 10.1111/j.1540-4560.2010.01670.x

[B24] Corr P. J. (2013). Approach and avoidance behaviour: multiple systems and their interactions. *Emot. Rev.* 5 285–290. 10.1177/1754073913477507

[B25] Crandall C. S. Miller J. M. White M. H. (2018). Changing norms following the 2016 U.S. Presidential Election: The Trump effect on prejudice. *Soc. Psycholog. Person. Sci.* 9 186–192. 10.1177/1948550617750735 41558976

[B26] Craske M. G. Treanor M. Conway C. C. Zbozinek T. Vervliet B. (2014). Maximizing exposure therapy: an inhibitory learning approach. *Behav. Res. Ther.* 58 10–23. 10.1016/j.brat.2014.04.006 24864005 PMC4114726

[B27] Cumsille P. E. Sayer A. G. Graham J. W. (2000). Perceived exposure to peer and adult drinking as predictors of growth in positive alcohol expectancies during adolescence. *J. Consult. Clin. Psychol.* 68 531–536. 10.1037/0022-006X.68.3.531 10883572

[B28] Davis T. Love B. C. Todd Maddox W. (2009). Anticipatory emotions in decision tasks: Covert markers of value or attentional processes? *Cognition* 112 195–200. 10.1016/j.cognition.2009.04.002 19428002 PMC2735832

[B29] Del Vicario M. Bessi A. Zollo F. Petroni F. Scala A. Caldarelli G. (2016). The spreading of misinformation online. *Proc. Natl. Acad. Sci. U. S. A.* 113 554–559. 10.1073/pnas.1517441113 26729863 PMC4725489

[B30] Den Ouden H. E. M. Kok P. de Lange F. P. (2012). How prediction errors shape perception, attention, and motivation. *Front. Psychol.* 3 1–12. 10.3389/fpsyg.2012.00548 23248610 PMC3518876

[B31] DeSteno D. Petty R. E. Wegener D. T. Rucker D. D. (2000). Beyond valence in the perception of likelihood: the role of emotion specificity. *J. Person. Soc. Psychol.* 78 397–416. 10.1037/0022-3514.78.3.397 10743870

[B32] DeYoung C. G. (2015). Cybernetic Big Five Theory. *J. Res. Person.* 56 33–58. 10.1016/j.jrp.2014.07.004

[B33] Dijksterhuis A. van Knippenberg A. Kruglanski A. W. Schaper C. (1996). Motivated social cognition: Need for closure effects on memory and judgment. *J. Exp. Soc. Psychol.* 32 254–270. 10.1006/jesp.1996.0012

[B34] Dort M. Strelow A. Schwinger M. Christiansen H. (2020a). What Teachers Think and Know about ADHD: Validation of the ADHD-school-expectation Questionnaire (ASE). *Intern. J. Dis. Dev. Educ.* 2020:1843142. 10.1080/1034912X.2020.1843142

[B35] Dort M. Strelow A. E. Schwinger M. Christiansen H. (2020b). Working with children with ADHD—a latent profile analysis of teachers’ and psychotherapists’ attitudes. *Sustainability* 12:9691. 10.3390/su12229691

[B36] Dunn J. R. Schweitzer M. E. (2005). Feeling and believing: The influence of emotion on trust. *J. Person. Soc. Psychol.* 88 736–748. 10.1037/0022-3514.88.5.736 15898872

[B37] Dunsmoor J. E. Paz R. (2015). Fear generalization and anxiety: Behavioral and neural mechanisms. *Biolog. Psychiatry* 78 336–343. 10.1016/j.biopsych.2015.04.010 25981173

[B38] Dymond S. Dunsmoor J. E. Vervliet B. Roche B. Hermans D. (2015). Fear generalization in humans: systematic review and implications for anxiety disorder research. *Behav. Ther.* 46 561–582. 10.1016/j.beth.2014.10.001 26459838

[B39] Esser J. K. (1998). Alive and well after 25 years: A review of groupthink research. *Org. Behav. Hum. Dec. Proc.* 73 116–141. 10.1006/obhd.1998.2758 9705799

[B40] Farrer C. Valentin G. Hupé J. M. (2013). The time windows of the sense of agency. *Consciou. Cogn.* 22 1431–1441. 10.1016/j.concog.2013.09.010 24161792

[B41] Feldman H. Friston K. J. (2010). Attention, uncertainty, and free-energy. *Front. Hum. Neurosci.* 4 1–23. 10.3389/fnhum.2010.00215 21160551 PMC3001758

[B42] Filipowicz A. Valadao D. Anderson B. Danckert J. (2018). Rejecting outliers: Surprising changes do not always improve belief updating. *Decision* 5 165–176. 10.1037/dec0000073

[B43] Friston K. (2009). The free-energy principle: a rough guide to the brain? *Trends Cogn. Sci.* 13 293–301. 10.1016/j.tics.2009.04.005 19559644

[B44] Friston K. Schwartenbeck P. FitzGerald T. Moutoussis M. Behrens T. Dolan R. J. (2013). The anatomy of choice: active inference and agency. *Front. Hum. Neurosci.* 7:598. 10.3389/fnhum.2013.00598 24093015 PMC3782702

[B45] Garrett N. Sharot T. (2017). Optimistic update bias holds firm: Three tests of robustness following Shah et al. *Consciousness Cogn.* 50 12–22. 10.1016/j.concog.2016.10.013 27836628 PMC5380127

[B46] Gasper K. Clore G. L. (2002). Attending to the big picture: Mood and global versus local processing of visual information. *Psycholog. Sci.* 13 34–40. 10.1111/1467-9280.00406 11892776

[B47] Geers A. L. Lassiter G. D. (2002). Effects of affective expectations on affective experience: The moderating role of optimism–pessimism. *Person. Soc. Psychol. Bull.* 28 1026–1039. 10.1177/01461672022811002

[B48] Geers A. L. Wellman J. A. Fowler S. L. Helfer S. G. France C. R. (2010). Dispositional optimism predicts placebo analgesia. *J. Pain* 11 1165–1171. 10.1016/j.jpain.2010.02.014 20627818 PMC2956003

[B49] Gershman S. J. (2015). A unifying probabilistic view of associative learning. *PLoS Comput. Biol.* 11 1–20. 10.1371/journal.pcbi.1004567 26535896 PMC4633133

[B50] Gershman S. J. Horvitz E. J. Tenenbaum J. B. (2015). Computational rationality: A converging paradigm for intelligence in brains, minds, and machines. *Science* 349 273–278. 10.1126/science.aac6076 26185246

[B51] Gollwitzer M. Thorwart A. Meissner K. (2018). Editorial: Psychological responses to violations of expectations. *Front. Psychol.* 8:2357. 10.3389/fpsyg.2017.02357 29410637 PMC5787099

[B52] Gottlieb J. Oudeyer P. Y. Lopes M. Baranes A. (2013). Information-seeking, curiosity, and attention: computational and neural mechanisms. *Trends Cogn. Sci.* 17 585–593. 10.1016/j.tics.2013.09.001 24126129 PMC4193662

[B53] Greve W. Wentura D. (2010). True lies: Self-stabilization without self-deception. *Consciousness Cogn.* 19 721–730. 10.1016/j.concog.2010.05.016 20646937

[B54] Griffiths M. D. (1994). The role of cognitive bias and skill in fruit machine gambling. *Br. J. Psychol.* 85 351–369. 10.1111/j.2044-8295.1994.tb02529.x

[B55] Griffiths O. Shehabi N. Murphy R. A. Le Pelley M. E. (2019). Superstition predicts perception of illusory control. *Br. J. Psychol.* 110 499–518. 10.1111/bjop.12344 30144046

[B56] Grupe D. W. Nitschke J. B. (2013). Uncertainty and anticipation in anxiety: An integrated neurobiological and psychological perspective. *Nat. Rev. Neurosci.* 14 488–501. 10.1038/nrn3524 23783199 PMC4276319

[B57] Hamamouche K. Keefe M. Jordan K. E. Cordes S. (2018). Cognitive load affects numerical and temporal judgments in distinct ways. *Front. Psychol.* 9:1783. 10.3389/fpsyg.2018.01783 30333769 PMC6176015

[B58] Hart W. Adams J. M. Alex Burton K. Shreves W. Hamilton J. C. (2012). Shaping reality vs. hiding from reality: reconsidering the effects of trait need for closure on information search. *J. Res. Person.* 46 489–496. 10.1016/j.jrp.2012.05.004

[B59] Haselgrove M. Aydin A. Pearce J. M. (2004). A partial reinforcement extinction effect despite equal rates of reinforcement during Pavlovian conditioning. *J. Exp. Psychol.* 30 240–250. 10.1037/0097-7403.30.3.240 15279514

[B60] Hashemi M. M. Gladwin T. E. de Valk N. M. Zhang W. Kaldewaij R. van Ast V. (2019). Neural dynamics of shooting decisions and the switch from freeze to fight. *Sci. Rep.* 9 1–10. 10.1038/s41598-019-40917-8 30862811 PMC6414631

[B61] Hawkins R. X. D. Goodman N. D. Goldstone R. L. (2019). The emergence of social norms and conventions. *Trends Cogn. Sci.* 23 158–169. 10.1016/j.tics.2018.11.003 30522867

[B62] Hechler T. Endres D. Thorwart A. (2016). Why harmless sensations might hurt in individuals with chronic pain: about heightened prediction and perception of pain in the mind. *Front. Psychol.* 7:1638. 10.3389/fpsyg.2016.01638 27826271 PMC5078757

[B63] Henslin J. M. (1967). Craps and magic. *Am. J. Soc.* 73 316–330. 10.1086/224479

[B64] Higgs S. (2015). Social norms and their influence on eating behaviours. *Appetite* 86 38–44. 10.1016/j.appet.2014.10.021 25451578

[B65] Hirsch C. R. Meeten F. Krahé C. Reeder C. (2016). Resolving ambiguity in emotional disorders: the nature and role of interpretation biases. *Annu. Rev. Clin. Psychol.* 12 281–305. 10.1146/annurev-clinpsy-021815-093436 27019398

[B66] Hirsh J. B. DeYoung C. G. Peterson J. B. (2009). Metatraits of the Big Five differentially predict engagement and restraint of behavior. *J. Person.* 77 1085–1102. 10.1111/j.1467-6494.2009.00575.x 19558442

[B67] Hoerger M. Quirk S. W. Chapman B. P. Duberstein P. R. (2012). Affective forecasting and self-rated symptoms of depression, anxiety, and hypomania: evidence for a dysphoric forecasting bias. *Cogn. Emot.* 26 1098–1106. 10.1080/02699931.2011.631985 22397734 PMC3371284

[B68] Hogg M. A. Hains S. C. (1998). Friendship and group identification: A new look at the role of cohesiveness in groupthink. *Eur. J. Soc. Psychol.* 28 323–341. 10.1002/(sici)1099-0992(199805/06)28:3<323::aid-ejsp854<3.3.co;2-p

[B69] Hohwy J. (2017). Priors in perception: Top-down modulation, Bayesian perceptual learning rate, and prediction error minimization. *Consciousness Cogn.* 47 75–85. 10.1016/j.concog.2016.09.004 27663763

[B70] Hoorens V. (2012). “Expectation,” in *Encyclopedia of Human Behavior*, 2nd Edn, ed. Ramachandran V. S. (Cambridge, MA: Academic Press), 142–149. 10.1016/B978-0-12-375000-6.00163-4

[B71] Huff N. C. LaBar K. S. (2010). “Generalization and specialization of conditioned learning,” in *Generalization of Knowledge* (1st Edn, eds Banich M. T. Caccamise D. (Hove: Psychology Press), 3–29. 10.4324/9780203848036

[B72] Jiang J. Summerfield C. Egner T. (2013). Attention sharpens the distinction between expected and unexpected percepts in the visual brain. *J. Neurosci.* 33 18438–18447. 10.1523/JNEUROSCI.3308-13.2013 24259568 PMC3834051

[B73] Johnson E. T. Tversky A. (1983). Affect, generalization, and the perception of risk. *J. Person. Soc. Psychol.* 45:20. 10.1037/0022-3514.45.1.20

[B74] Johnson-Laird P. N. Khemlani S. S. Goodwin G. P. (2015). Logic, probability, and human reasoning. *Trends Cogn. Sci.* 19 201–214. 10.1016/j.tics.2015.02.006 25770779

[B75] Kappes A. Harvey A. H. Lohrenz T. Montague P. R. Sharot T. (2020). Confirmation bias in the utilization of others’ opinion strength. *Nat. Neurosci.* 23 130–137. 10.1038/s41593-019-0549-2 31844311

[B76] Karsh N. Eitam B. (2015). I control therefore I do: judgments of agency influence action selection. *Cognition* 138 122–131. 10.1016/j.cognition.2015.02.002 25724007

[B77] Kastner-Dorn A. K. Andreatta M. Pauli P. Wieser M. J. (2018). Hypervigilance during anxiety and selective attention during fear: using steady-state visual evoked potentials (ssVEPs) to disentangle attention mechanisms during predictable and unpredictable threat. *Cortex* 106 120–131. 10.1016/j.cortex.2018.05.008 29929061

[B78] Kauff M. Asbrock F. Thörner S. Wagner U. (2013). Side effects of multiculturalism: The interaction effect of a multicultural ideology and authoritarianism on prejudice and diversity beliefs. *Person. Soc. Psychol. Bull.* 39 305–320. 10.1177/0146167212473160 23344162

[B79] Kemmelmeier M. (2015). The closed-mindedness that wasn’t: Need for structure and expectancy-inconsistent information. *Front. Psychol.* 6:896. 10.3389/fpsyg.2015.00896 26191017 PMC4488610

[B80] Klayman J. Ha Y.-W. (1987). Confirmation, disconfirmation, and information in hypothesis testing. *Psychol. Rev.* 94 211–228. 10.1037//0033-295x.94.2.211

[B81] Klein S. B. Cosmides L. Tooby J. Chance S. (2002). Decisions and the evolution of memory: Multiple systems, multiple functions. *Psycholog. Rev.* 109 306–329. 10.1037/0033-295X.109.2.306 11990320

[B82] Knopp B. Velychko D. Dreibrodt J. Schütz A. C. Endres D. (2020). “Evaluating perceptual predictions based on movement primitive models in VR- and online-experiments,” in *ACM SAP* 20 1–9. 10.1145/3385955.3407940

[B83] Koenig S. Uengoer M. Lachnit H. (2017). Attentional bias for uncertain cues of shock in human fear conditioning: evidence for attentional learning theory. *Front. Hum. Neurosci.* 11 1–13. 10.3389/fnhum.2017.00266 28588466 PMC5440506

[B84] Kok P. Rahnev D. Jehee J. F. M. Lau H. C. De Lange F. P. (2012). Attention reverses the effect of prediction in silencing sensory signals. *Cerebral Cortex* 22 2197–2206. 10.1093/cercor/bhr310 22047964

[B85] Körfer K. Schemer L. Kube T. Glombiewski J. A. (2020). An experimental analogue study on the “dose-response relationship” of different therapeutic instructions for pain exposures: The more, the better? *J. Pain Res.* 13 3181–3193. 10.2147/JPR.S265709 33293855 PMC7719044

[B86] Korn C. W. Prehn K. Park S. Q. Walter H. Heekeren H. R. (2012). Positively biased processing of self-relevant social feedback. *J. Neurosci.* 32 16832–16844. 10.1523/JNEUROSCI.3016-12.2012 23175836 PMC6621762

[B87] Kotzur P. F. Wagner U. (2021). The dynamic relationship between contact opportunities, positive and negative intergroup contact, and prejudice: a longitudinal investigation. *J. Person. Soc. Psychol.* 120 418–442. 10.1037/pspi0000258 32700961

[B88] Krause E. Benke C. Koenig J. Thayer J. F. Hamm A. O. Pané-Farré C. A. (2018). Dynamics of defensive response mobilization to approaching external versus interoceptive threat. *Biolog. Psychiatry* 3 525–538. 10.1016/j.bpsc.2017.12.002 29884283

[B89] Kress L. Aue T. (2017). The link between optimism bias and attention bias: A neurocognitive perspective. *Neurosci. Biobehav. Rev.* 80 688–702. 10.1016/j.neubiorev.2017.07.016 28780313

[B90] Kruglanski A. W. Jasko K. Friston K. (2020). All thinking is ‘wishful’ thinking. *Trends Cogn. Sci.* 24 413–424. 10.1016/j.tics.2020.03.004 32284177

[B91] Kruglanski A. W. Webster D. M. (1996). Motivated closing of the mind: “Seizing” and “freezing.”. *Psychol. Rev.* 103 263–283. 10.1037/0033-295X.103.2.263 8637961

[B92] Kruschke J. K. (2008). Bayesian approaches to associative learning: From passive to active learning. *Learn. Behav.* 36 210–226. 10.3758/LB.36.3.210 18683466

[B93] Kube T. Rief W. Glombiewski J. A. (2017). On the maintenance of expectations in major depression - Investigating a neglected phenomenon. *Front. Psychol.* 8 1–7. 10.3389/fpsyg.2017.00009 28149287 PMC5241292

[B94] Kube T. Rief W. Gollwitzer M. Gärtner T. Glombiewski J. A. (2019). Why dysfunctional expectations in depression persist - Results from two experimental studies investigating cognitive immunization. *Psycholog. Med.* 49 1532–1544. 10.1017/S0033291718002106 30131084

[B95] Kube T. Schwarting R. Rozenkrantz L. Glombiewski J. A. Rief W. (2020). Distorted cognitive processes in major depression: a predictive processing perspective. *Biol. Psychiatry* 87 388–398. 10.1016/j.biopsych.2019.07.017 31515055

[B96] Kuntsche E. Gabhainn S. N. Roberts C. Windlin B. Vieno A. Bendtsen P. (2014). Drinking motives and links to alcohol use in 13 European countries. *J. Stud. Alcohol Drugs* 75 428–437. 10.15288/jsad.2014.75.428 24766755

[B97] Kutlu M. G. Schmajuk N. A. (2012). Solving Pavlov’s puzzle: Attentional, associative, and flexible configural mechanisms in classical conditioning. *Learn. Behav.* 40 269–291. 10.3758/s13420-012-0083-5 22927001

[B98] Kwisthout J. Bekkering H. van Rooij I. (2017). To be precise, the details don’t matter: On predictive processing, precision, and level of detail of predictions. *Brain Cogn.* 112 84–91. 10.1016/j.bandc.2016.02.008 27114040

[B99] LeDoux J. E. Moscarello J. Sears R. Campese V. (2017). The birth, death and resurrection of avoidance: A reconceptualization of a troubled paradigm. *Mole. Psychiatry* 22 24–36. 10.1038/mp.2016.166 27752080 PMC5173426

[B100] Lee M. D. Zhang S. Munro M. Steyvers M. (2011). Psychological models of human and optimal performance in bandit problems. *Cogn. Syst. Res.* 12 164–174. 10.1016/j.cogsys.2010.07.007

[B101] Legros S. Cislaghi B. (2020). Mapping the social-norms literature: An overview of reviews. *Perspect. Psycholog. Sci.* 15 62–80. 10.1177/1745691619866455 31697614 PMC6970459

[B102] Liebe U. Meyerhoff J. Kroesen M. Chorus C. Glenk K. (2018). From welcome culture to welcome limits? Uncovering preference changes over time for sheltering refugees in Germany. *PLoS One* 13 1–13. 10.1371/journal.pone.0199923 30067769 PMC6070168

[B103] Lommen M. J. J. Engelhard I. M. van den Hout M. A. (2010). Neuroticism and avoidance of ambiguous stimuli: Better safe than sorry? *Person. Indiv. Diff.* 49 1001–1006. 10.1016/j.paid.2010.08.012

[B104] Lovibond P. F. Mitchell C. J. Minard E. Brady A. Menzies R. G. (2009). Safety behaviours preserve threat beliefs: Protection from extinction of human fear conditioning by an avoidance response. *Behav. Res. Ther.* 47 716–720. 10.1016/j.brat.2009.04.013 19457472

[B105] Lucke S. Lachnit H. Stüttgen M. C. Uengoer M. (2014). The impact of context relevance during extinction learning. *Learn. Behav.* 42 256–269. 10.3758/s13420-014-0143-0 24934214

[B106] Mäki-Marttunen V. Hagen T. Espeseth T. (2019). Task context load induces reactive cognitive control: An fMRI study on cortical and brain stem activity. *Cogn. Affect. Behav. Neurosci.* 19 945–965. 10.3758/s13415-019-00691-6 30659515 PMC6711881

[B107] Marroquín B. Boyle C. C. Nolen-Hoeksema S. Stanton A. L. (2016). Using emotion as information in future-oriented cognition: individual differences in the context of state negative affect. *Personal. Indiv. Diff.* 95 121–126. 10.1016/j.paid.2016.02.033. 27041783 PMC4811627

[B108] Marroquín B. Nolen-Hoeksema S. (2015). Event prediction and affective forecasting in depressive cognition: using emotion as information about the future. *J. Soc. Clin. Psychol.* 34 117–134. 10.1521/jscp.2015.34.2.117 26146452 PMC4486478

[B109] McClelland J. L. McNaughton B. L. O’Reilly R. C. (1995). Why there are complementary learning systems in the hippocampus and neocortex: Insights from the successes and failures of connectionist models of learning and memory. *Psychol. Rev.* 102 419–457. 10.1037/0033-295X.102.3.419 7624455

[B110] McNamee D. Wolpert D. M. (2019). Internal models in biological control. *Annu. Rev. Control Robot. Auton. Syst.* 2 339–364. 10.1146/annurev-control-060117-105206 31106294 PMC6520231

[B111] Mehlhorn K. Newell B. R. Todd P. M. Lee M. D. Morgan K. Braithwaite V. A. (2015). Unpacking the exploration-exploitation tradeoff: A synthesis of human and animal literatures. *Decision* 2 191–215. 10.1037/dec0000033

[B112] Meltzoff A. N. Moore M. K. (1997). Explaining facial imitation. A theoretical model. *Early Dev. Parent.* 6 179–192. 10.1002/(SICI)1099-0917(199709/12)6:3/4<179::AID-EDP157<3.0.CO;2-R 24634574 PMC3953219

[B113] Mickley Steinmetz K. R. Schmidt K. Zucker H. R. Kensinger E. A. (2012). The effect of emotional arousal and retention delay on subsequent-memory effects. *Cogn. Neurosci.* 3 150–159. 10.1080/17588928.2012.677421 24171733 PMC3818726

[B114] Miller D. T. Turnbull W. (1986). Expectancies and interpersonal processes. *Ann. Rev. Psychol.* 37 233–256. 10.1146/annurev.psych.37.1.233

[B115] Miller G. (2007). Mental health and the mass media: room for improvement. *Lancet* 370 1015–1016. 10.1016/S0140-6736(07)61246-X 17804055

[B116] Mirza M. B. Adams R. A. Mathys C. Friston K. J. (2018). Human visual exploration reduces uncertainty about the sensed world. *PLoS One* 13 1–20. 10.1371/journal.pone.0190429 29304087 PMC5755757

[B117] Miskovic V. Keil A. (2012). Acquired fears reflected in cortical sensory processing: A review of electrophysiological studies of human classical conditioning. *Psychophysiology* 49 1230–1241. 10.1111/j.1469-8986.2012.01398.x 22891639 PMC3422776

[B118] Moore J. W. (2016). What is the sense of agency and why does it matter? *Front. Psychol.* 7 1–9. 10.3389/fpsyg.2016.01272 27621713 PMC5002400

[B119] Morton D. L. El-Deredy W. Morton A. S. Elliott R. Jones A. K. P. (2011). Optimism facilitates the utilisation of prior cues. *Eur. J. Personal.* 25 424–430. 10.1002/per.805

[B120] Mowrer O. H. (1956). Two-factor learning theory reconsidered, with special reference to secondary reinforcement and the concept of habit. *Psychol. Rev.* 63 114–128. 10.1037/h0040613 13310707

[B121] Mueller E. M. Burgdorf C. Chavanon M. L. Schweiger D. Wacker J. Stemmler G. (2014). Dopamine modulates frontomedial failure processing of agentic introverts versus extraverts in incentive contexts. *Cogn. Affect. Behav. Neurosci.* 14 756–768. 10.3758/s13415-013-0228-9 24323704

[B122] Mueller E. M. Makeig S. Stemmler G. Hennig J. Wacker J. (2011). Dopamine effects on human error processing depend on Catechol-O-Methyltransferase VAL158MET genotype. *J. Neurosci.* 31 15818–15825. 10.1523/JNEUROSCI.2103-11.2011 22049425 PMC6623004

[B123] Mueller E. M. Panitz C. Pizzagalli D. A. Hermann C. Wacker J. (2015). Midline theta dissociates agentic extraversion and anhedonic depression. *Personal. Indiv. Diff.* 79 172–177. 10.1016/j.paid.2014.10.043

[B124] Neuberg S. L. Judice T. N. West S. G. (1997). What the need for closure scale measures and what it does not: Toward differentiating among related epistemic motives. *Personality Proc. Indiv. Diff.* 72 1396–1412. 10.1037/0022-3514.72.6.1396

[B125] Neuberg S. L. Newsom J. T. (1993). Personal need for structure: Individual differences in the desire for simple structure. *J. Person. Soc. Psychol.* 65 113–131. 10.1037/0022-3514.65.1.113

[B126] Nickerson R. S. (1998). Confirmation bias: A ubiquitous phenomenon in many guises. *Rev. Gen. Psychol.* 2 175–220. 10.1037/1089-2680.2.2.175

[B127] Niv Y. (2019). Learning task-state representations. *Nat. Neurosci.* 22 1544–1553. 10.1038/s41593-019-0470-8 31551597 PMC7241310

[B128] Ogan C. Willnat L. Pennington R. Bashir M. (2014). The rise of anti-Muslim prejudice: Media and Islamophobia in Europe and the United States. *Internat. Comm. Gazette* 76 27–46. 10.1177/1748048513504048

[B129] Oken B. S. Salinsky M. (1992). Alertness and attention: basic science and electrophysiologic correlates. *J. Clin. Neurophys.* 9 480–494. 10.1097/00004691-199210000-00003 1361195

[B130] Oliver R. L. (1980). A cognitive model of the antecedents and consequences of satisfaction decisions. *J. Market. Res.* 17:460. 10.2307/3150499

[B131] Oliver R. L. (2014). *Satisfaction: A Behavioral Perspective on the Consumer.* Milton Park: Routledge.

[B132] Olsson A. Phelps E. A. (2004). Learned fear of “unseen” faces after Pavlovian, observational, and instructed fear. *Psychol. Sci.* 15 822–828. 10.1111/j.0956-7976.2004.00762.x 15563327

[B133] Panitz C. Keil A. Mueller E. M. (2019). Extinction-resistant attention to long-term conditioned threat is indexed by selective visuocortical alpha suppression in humans. *Sci. Rep.* 9:15809. 10.1038/s41598-019-52315-1 31676781 PMC6825167

[B134] Paulus A. Rohr M. Dotsch R. Wentura D. (2016). Positive feeling, negative meaning: Visualizing the mental representations of in-group and out-group smiles. *PLoS One* 11 1–18. 10.1371/journal.pone.0151230 26963621 PMC4786158

[B135] Pearce J. M. Hall G. (1980). A model for Pavlovian learning: variations in the effectiveness of conditioned but not of unconditioned stimuli. *Psycholog. Rev.* 87 532–552. 10.1037/0033-295X.87.6.532 7443916

[B136] Pearl R. L. Puhl R. M. Brownell K. D. (2012). Positive media portrayals of obese persons: Impact on attitudes and image preferences. *Health Psychol.* 31 821–829. 10.1037/a0027189 22309884

[B137] Pepperdine E. Lomax C. Freeston M. H. (2018). Disentangling intolerance of uncertainty and threat appraisal in everyday situations. *J. Anxiety Dis.* 57 31–38. 10.1016/j.janxdis.2018.04.002 29724665

[B138] Petersen S. E. Posner M. I. (2012). The attention system of the human brain: 20 years after. *Annu. Rev. Neurosci.* 35 73–89. 10.1146/annurev-neuro-062111-150525 22524787 PMC3413263

[B139] Piaget J. (1952). *The Origins of Intelligence in Children.* New York, NY: International Universities Press.

[B140] Pinquart M. Block H. (2020). Coping with broken achievement-related expectations in students from elementary school: an experimental study. *Intern. J. Dev. Sci.* 14 9–17. 10.3233/DEV-200001

[B141] Pinquart M. Endres D. Teige-Mocigemba S. Panitz C. Schütz A. C. (2021a). Why expectations do or do not change after expectation violation: a comparison of seven models. *Conscious. Cogn.* 89:103086. 10.1016/j.concog.2021.103086 33550190

[B142] Pinquart M. Rothers A. Gollwitzer M. Khosrowtaj Z. Pietzsch M. Panitz C. (2021b). Predictors of coping with expectation violation: an integrative review. *Rev. Gen. Psychol.* 25 321–333. 10.1177/10892680211024123

[B143] Proulx T. Inzlicht M. (2012). The five “A”s of meaning maintenance: Finding meaning in the theories of sense-making. *Psycholog. Inq.* 23 317–335. 10.1080/1047840X.2012.702372

[B144] Proulx T. Inzlicht M. Harmon-Jones E. (2012). Understanding all inconsistency compensation as a palliative response to violated expectations. *Trends Cogn. Sci.* 16 285–291. 10.1016/j.tics.2012.04.002 22516239

[B145] Rescorla R. A. (1969). Pavlovian conditioned inhibition. *Psycholog. Bull.* 72 77–94. 10.1037/h0027760

[B146] Rescorla R. A. Wagner A. R. (1972). “A theory of Pavlovian conditioning: Variations in the effectiveness of reinforcement and nonreinforcement,” in *Classical Conditioning II: Current Research and Theory*, eds Black A. H. Prokasy W. F. (New York, NY: Appleton-Century-Crofts), 64–99.

[B147] Rief W. Glombiewski J. A. Gollwitzer M. Schubö A. Schwarting R. Thorwart A. (2015). Expectancies as core features of mental disorders. *Curr. Opin. Psychiatry* 28 378–385. 10.1097/YCO.0000000000000184 26164612

[B148] Riek B. M. Mania E. W. Gaertner S. L. (2013). Reverse subtyping: the effects of prejudice level on the subtyping of counterstereotypic outgroup members. *Basic Appl. Soc. Psychol.* 35 409–417. 10.1080/01973533.2013.823616

[B149] Robertson K. Tustin K. (2018). Students who limit their drinking, as recommended by national guidelines, are stigmatized, ostracized, or the subject of peer pressure: Limiting consumption is all but prohibited in a culture of intoxication. *Subst. Abuse: Res. Treat.* 12:1178221818792414. 10.1177/1178221818792414 30093798 PMC6081750

[B150] Roese N. J. Sherman J. W. (2007). “Expectancy,” in *Social Psychology: Handbook of Basic Principles*, 2nd Edn, eds Higgins E. T. Kruglanski A. W. (New York, NY: Guilford Press), 91–115.

[B151] Romer D. Hennessy M. (2007). A biosocial-affect model of adolescent sensation seeking: The role of affect evaluation and peer-group influence in adolescent drug use. *Prevent. Sci.* 8 89–101. 10.1007/s11121-007-0064-7 17286212

[B152] Rotter J. B. (1966). Generalized expectancies for internal versus external control of reinforcement. *Psycholog. Monogr. Gen. Appl.* 80 1–28. 10.1037/h0092976 5340840

[B153] Rubo M. Huestegge L. Gamer M. (2020). Social anxiety modulates visual exploration in real life – but not in the laboratory. *Br. J. Psychol.* 111 233–245. 10.1111/bjop.12396 30945279 PMC7187184

[B154] Sarathchandra D. Haltinner K. (2020). Trust/distrust judgments and perceptions of climate science: A research note on skeptics’ rationalizations. *Public Understand. Sci.* 29 53–60. 10.1177/0963662519886089 31691642

[B155] Savage S. W. Spano L. P. Bowers A. R. (2019). The effects of age and cognitive load on peripheral-detection performance. *J. Vis.* 19 1–17. 10.1167/19.1.15 PMC6348997 30677125

[B156] Scheier M. F. Carver C. S. (1985). Optimism, coping, and health: Assessment and implications of generalized outcome expectancies. *Health Psychol.* 4 219–247. 10.1037/0278-6133.4.3.219 4029106

[B157] Scherer A. M. Windschitl P. D. O’Rourke J. Smith A. R. (2012). Hoping for more: The influence of outcome desirability on information seeking and predictions about relative quantities. *Cognition* 125 113–117. 10.1016/j.cognition.2012.06.013 22832177

[B158] Schultz W. Dayan P. Montague P. R. (1997). A neural substrate of prediction and reward. *Science* 275 1593–1599. 10.1126/science.275.5306.1593 9054347

[B159] Schwarz N. Clore G. L. (2003). Mood as Information: 20 Years Later. *Psychol. Inq.* 14 296–303. 10.1080/1047840x.2003.9682896

[B160] Schween R. Hegele M. (2017). Feedback delay attenuates implicit but facilitates explicit adjustments to a visuomotor rotation. *Neurobiol. Learn. Memory* 140 124–133. 10.1016/j.nlm.2017.02.015 28257877

[B161] Seligman M. E. P. (1972). Learned helplessness. *Annu. Rev. Med.* 23 407–412. 10.1146/annurev.me.23.020172.002203 4566487

[B162] Shadmehr R. Smith M. A. Krakauer J. W. (2010). Error correction, sensory prediction, and adaptation in motor control. *Annu. Rev. Neurosci.* 33 89–108. 10.1146/annurev-neuro-060909-153135 20367317

[B163] Sharot T. Korn C. W. Dolan R. J. (2011). How unrealistic optimism is maintained in the face of reality. *Nat. Neurosci.* 14 1475–1479. 10.1038/nn.2949 21983684 PMC3204264

[B164] Sharot T. Phelps E. A. (2004). How arousal modulates memory: disentangling the effects of attention and retention. *Cogn. Affect. Behav. Neurosci.* 4 294–306. 10.3758/CABN.4.3.294 15535165

[B165] Sharot T. Sunstein C. R. (2020). How people decide what they want to know. *Nat. Hum. Behav.* 4 14–19. 10.1038/s41562-019-0793-1 31932690

[B166] Sherman J. W. Lee A. Y. Bessenoff G. R. Frost L. A. (1998). Stereotype efficiency reconsidered: Encoding flexibility under cognitive load. *J. Person. Soc. Psychol.* 75 589–606. 10.1037/0022-3514.75.3.589 9781404

[B167] Smout C. A. Tang M. F. Garrido M. I. Mattingley J. B. (2019). Attention promotes the neural encoding of prediction errors. *PLoS Biol.* 17:1–22. 10.1371/journal.pbio.2006812 30811381 PMC6411367

[B168] Soral W. Kofta M. Bukowski M. (2021). Helplessness experience and intentional (un-)binding: Control deprivation disrupts the implicit sense of agency. *J. Exp. Psychol.* 150 289–305. 10.1037/xge0000791 32658528

[B169] Sosa R. Ramírez M. N. (2019). Conditioned inhibition: Historical critiques and controversies in the light of recent advances. *J. Exp. Psychol.* 45 17–42. 10.1037/xan0000193 30604993

[B170] Spicer S. G. Mitchell C. J. Wills A. J. Jones P. M. (2020). Theory protection in associative learning: Humans maintain certain beliefs in a manner that violates prediction error. *J. Exp. Psychol.* 46 151–161. 10.1037/xan0000225 31556642

[B171] Steinke J. (2017). Adolescent girls’ STEM identity formation and media images of STEM professionals: Considering the influence of contextual cues. *Front. Psychol.* 8 1–15. 10.3389/fpsyg.2017.00716 28603505 PMC5445165

[B172] Stürmer S. Rohmann A. Froehlich L. van der Noll J. (2019). Muslim immigration, critical events, and the seeds of majority members’ support for radical responses: An interactionist perspective. *Personal. Soc. Psychol. Bull.* 45 133–145. 10.1177/0146167218780989 29911500

[B173] Summerfield C. Tsetsos K. (2015). Do humans make good decisions? *Trends Cogn. Sci.* 19 27–34. 10.1016/j.tics.2014.11.005 25488076 PMC4286584

[B174] Sutton R. S. Barto A. G. (1998). *Introduction to reinforcement learning.* Cambridge, MA: MIT Press.

[B175] Sweeny K. Dillard A. (2014). The effects of expectation disconfirmation on appraisal, affect, and behavioral intentions. *Risk Anal.* 34 711–720. 10.1111/risa.12129 24151990

[B176] ter Bogt T. F. M. Engels R. C. M. E. Bogers S. Kloosterman M. (2010). “Shake it baby, shake it”: media preferences, sexual attitudes and gender stereotypes among adolescents. *Sex Roles* 63 844–859. 10.1007/s11199-010-9815-1 21212809 PMC2993884

[B177] Thompson S. C. Armstrong W. Thomas C. (1998). Illusions of control, underestimations, and accuracy: A control heuristic explanation. *Psycholog. Bull.* 123 143–161. 10.1037/0033-2909.123.2.143 9522682

[B178] Todorov E. Jordan M. I. (2002). Optimal feedback control as a theory of motor coordination. *Nat. Neurosci.* 5 1226–1235. 10.1038/nn963 12404008

[B179] Torrents-Rodas D. Koenig S. Uengoer M. Lachnit H. (2021). A rise in prediction error increases attention to irrelevant cues. *Biolog. Psychol.* 159:108007. 10.1016/j.biopsycho.2020.108007 33321151

[B180] Uengoer M. Lissek S. Tegenthoff M. Manahan-Vaughan D. Lachnit H. (2020a). Principles of extinction learning of nonaversive experience. *Neuroforum* 26 151–159. 10.1515/nf-2020-0013

[B181] Uengoer M. Thorwart A. Lucke S. Wöhr M. Lachnit H. (2020b). Adding or removing context components equally disrupts extinction in human predictive learning. *Behav. Proc.* 179 104216. 10.1016/j.beproc.2020.104216 32771411

[B182] Unkelbach C. Forgas J. P. Denson T. F. (2008). The turban effect: The influence of Muslim headgear and induced affect on aggressive responses in the shooter bias paradigm. *J. Exp. Soc. Psychol.* 44 1409–1413. 10.1016/j.jesp.2008.04.003

[B183] Urbán R. (2009). Smoking outcome expectancies mediate the association between sensation seeking, peer smoking, and smoking among young adolescents. *Nicot. Tob. Res.* 12 59–68. 10.1093/ntr/ntp174 19959571 PMC2802571

[B184] Vlaeyen J. W. S. Linton S. J. (2012). Fear-avoidance model of chronic musculoskeletal pain: 12 years on. *Pain* 153 1144–1147. 10.1016/j.pain.2011.12.009 22321917

[B185] Vogt J. De Houwer J. Koster E. H. W. Van Damme S. Crombez G. (2008). Allocation of spatial attention to emotional stimuli depends upon arousal and not valence. *Emotion* 8 880–885. 10.1037/a0013981 19102600

[B186] Wieser M. J. Reicherts P. Juravle G. von Leupoldt A. (2016). Attention mechanisms during predictable and unpredictable threat — A steady-state visual evoked potential approach. *NeuroImage* 139 167–175. 10.1016/j.neuroimage.2016.06.026 27318217

[B187] Willadsen M. Best L. M. Wöhr M. Clarke P. B. S. (2018). Effects of anxiogenic drugs on the emission of 22- and 50-kHz ultrasonic vocalizations in adult rats. *Psychopharmacology* 235 2435–2445. 10.1007/s00213-018-4942-4 29909426

[B188] Wilson T. D. Dunn E. W. (2004). Self-knowledge: Its limits, value, and potential for improvement. *Ann. Rev. Psychol.* 55 493–518. 10.1146/annurev.psych.55.090902.141954 14744224

[B189] Yacubian J. Gläscher J. Schroeder K. Sommer T. Braus D. F. Büchel C. (2006). Dissociable systems for gain- and loss-related value predictions and errors of prediction in the human brain. *J. Neurosci.* 26 9530–9537. 10.1523/JNEUROSCI.2915-06.2006 16971537 PMC6674602

[B190] Yarritu I. Matute H. Vadillo M. A. (2014). Illusion of control: The role of personal involvement. *Exp. Psychol.* 61 38–47. 10.1027/1618-3169/a000225 23948387 PMC4013923

[B191] Zhao Y. Zhang J. (2017). Consumer health information seeking in social media: a literature review. *Health Inform. Lib. J.* 34 268–283. 10.1111/hir.12192 29045011

